# Integrated Single-Cell and Bulk Transcriptomics Unveils Immune Profiles in Chick Erythroid Cells upon Avian Pathogenic *Escherichia coli* Infection

**DOI:** 10.3390/ani16020179

**Published:** 2026-01-07

**Authors:** Fujuan Cai, Xianjue Wang, Chunzhi Wang, Yuzhen Wang, Wenguang Zhang

**Affiliations:** 1 School of Life Sciences, Inner Mongolia Agricultural University, Hohhot 010018, China; caifujuan@163.com (F.C.); xianjuewang@163.com (X.W.); 2 Inner Mongolia Key Laboratory of Biomanufacturing Technology, Hohhot 010018, China; 3 School of Statistics and Mathematics, Inner Mongolia University of Finance and Economics, Hohhot 010070, China; nmwcz@126.com

**Keywords:** single-cell RNA-Seq, erythroid cells, chick, immune profiles, APEC

## Abstract

Nucleated erythroid cells (NECs) have emerged as active participants in immune responses in addition to their canonical oxygen transport function. The subpopulations and immune heterogeneity of chick erythroid cells (ch-ECs) upon infection have not been fully characterized. Single-cell RNA sequencing (scRNA-seq) was used to profile ch-ECs in chicks infected with avian pathogenic *Escherichia coli* (APEC). Unsupervised clustering uncovered ten distinct ch-EC subpopulations (C1–C10), with significant compositional shifts between infected and control groups. Pseudotime analysis revealed a developmental continuum: C1, C3, C5, and C9 as early progenitors; C2, C4, C6, C7, and C10 as mature erythroid cells; and C8 as a naive population. We revealed 62 immune-related genes, including protein kinases and heat shock proteins, and subpopulation-specific differentially expressed genes (DEGs) linked to immune functions. SCENIC analysis revealed Fos, Srf, and Stat3 as key transcription factors with elevated regulon activity and specificity following infection. Subpopulations C2, C4, C6, and C7, which exhibited marked abundance changes, were scrutinized for immune relevance through integrated multi-omics analysis. Immune-related genes including *FOS*, *AKAP9*, *HS6ST1*, *GAB3*, *TFRC*, *HSPA8*, *HSP90AA1*, and *DNAJB6* were identified. Enrichment analysis indicated activation of the MHC class I antigen presentation pathway, while pathways such as Mitogen-Activated Protein Kinase (MAPK) signaling, NOD-like receptor (NLR) signaling, and the heat shock response were found to be suppressed. In conclusion, this study delineates the immune gene repertoire and signaling networks of ch-ECs during APEC infection, offering new perspectives on NEC immunoregulatory functions.

## Simple Summary

In addition to their primary role in oxygen transport, chicken erythroid cells have been shown to participate in immune responses. This study utilized advanced single-cell analysis alongside bulk RNA sequencing to explore the immune mechanisms of erythroid cells in chicks infected with avian pathogenic *Escherichia coli* (APEC). Through integrated multi-omics analysis, we identified immune-related genes and signaling pathways associated with antiviral defense. Preliminary single-cell analysis revealed heterogeneity within the erythroid cell population, unveiling four erythrocyte subtypes that exhibited significant quantitative changes following APEC infection, as well as key transcription factors potentially involved in pathogen defense. This research represents the first systematic characterization of the immune landscape of chick erythroid cells during infection, enhancing our understanding of avian immune mechanisms and providing a theoretical foundation for the development of novel disease-resistance strategies in poultry, thereby contributing to sustainable poultry production.

## 1. Introduction

Erythroid cells (ECs), the primary cellular component of blood, constitute approximately 70% of all cells in the adult human body [1]. Beyond their well-established role in oxygen transport, emerging evidence highlights the immunomodulatory functions of nucleated erythroid cells (NECs). Human NECs exhibit immunosuppressive activity via arginase-2 and reactive oxygen species (ROS) production [2]. Additionally, mammalian (human and mouse) ECs express Toll-like receptor (TLR) 9, which binds bacterial CpG-DNA, mitochondrial DNA, and plasmacytoid cell-derived DNA to enhance phagocytosis and immune activation [3]. Notably, chicken erythroid cells (ch-ECs) rapidly activate complement genes upon *Escherichia coli* (*E. coli*) adhesion [4]. Goose ECs demonstrate bacterial adhesion and phagocytic capacity [5]. Hence, exploring the immunological roles of ECs carries significant mechanistic implications.

Avian ECs retain their nuclei throughout their life cycle. The expression of *IL-6* and *IL-8* was upregulated in goose ECs following *E. coli* stimulation [5]. Ch-ECs exhibit pathogen-specific responses, as demonstrated by the increased expression of interferon *(IFN)-α* and *IL-8* mRNA upon TLR3 activation by polyinosinic: polycytidylic acid (poly(I:C)) [6]. However, research on the immune functions of avian ECs remains limited.

Single-cell RNA sequencing (scRNA-seq) serves as a high-resolution platform for investigating ontogeny and diseases, dissecting cellular heterogeneity [7], lineage trajectories [8], and transcriptomic signatures. ScRNA-seq has been widely adopted to investigate diverse biological processes, including NDV infection responses in chicken lung cells and fibroblasts [9] and late-stage embryonic liver dynamics [10]. Beyond avian systems, scRNA-seq has also advanced our understanding of nucleated ECs functions, such as human NECs [11] and fish ECs [12]. However, the immune role of ch-ECs remains unexplored. Our study employed scRNA-seq to analyze changes in ch-EC subpopulations and their functional dynamics following chicks infected with avian pathogenic *Escherichia coli* (APEC), thereby establishing a foundation for understanding the immunological functions of ECs.

The immunobiology of ch-ECs at the single-cell level is not well understood. In this study, we combined exploratory scRNA-seq with bulk RNA-seq and RT-qPCR validation to analyze the ch-ECs immune response 12 h post-APEC infection. Single-cell data identify cell heterogeneity and candidate genes, while bulk RNA-seq and RT-qPCR provide population-level insights.

## 2. Materials and Methods

### 2.1. Ethics Statement

All animal experimental procedures were approved by the Animal Ethics Committee of Inner Mongolia Agricultural University (Approval No. NND2022119) and were conducted in strict accordance with institutional and national guidelines for the care and use of animals. The study was performed at the Molecular Immunology Laboratory, College of Life Sciences, Inner Mongolia Agricultural University.

### 2.2. Animals and Experimental Groups

A total of 200 healthy Hongyu 380 broilers were obtained from the Shandong Jining Hongyu 380 Hatchery (Yanzhou District, Jining City, Shandong Province, China). To avoid potential immunosuppressive effects linked to the excessive secretion of male hormones in roosters and to maintain gender homogeneity among experimental subjects, this study exclusively utilized female chickens for all procedures. All chicks were maintained under standardized conditions, with ambient temperature within the poultry thermoneutral zone (20–24 °C) and a 16 h light/8 h dark cycle to support natural behavioral rhythms. Feed was provided three times daily, with ad libitum access to both feed and water. Twenty 10-day-old chicks were randomly assigned to two groups: control (CON) and APEC-infected. Drawing upon previous literature reviews and initial experimental findings, a 12 h post-infection time point was identified as the critical observation window for this study. The detailed treatment protocol is outlined as follows: chicks in the CON group (*n* = 10) received an intramuscular injection of 200 μL PBS, while those in the APEC-infected group (*n* = 10) were injected intramuscularly with 200 μL of APEC at a concentration of 3 × 10^9^ CFU/mL (as determined by pilot experiments).

### 2.3. Sample Collection

Peripheral blood was collected from independent chicks designated for each phase. Ch-ECs were isolated by density gradient centrifugation, as previously described [5]. Briefly, blood was drawn from the wing vein using a sterile syringe containing 0.1 mL/mL EDTA·2K (10×) and centrifuged at 2500× *g* for 10 min to separate the cellular components. The pelleted blood cells were resuspended in 0.9% NaCl solution and layered onto a 51% Percoll (Cytiva, Uppsala, Sweden) density gradient. After 30 min of phase separation at room temperature, gradient centrifugation was performed at 500× *g* for 30 min, followed by three washes with 0.9% NaCl solution to obtain ch-ECs for subsequent transcriptomic analyses. Concurrently, tissue samples from the heart, liver, spleen, lungs, and kidneys (*n* = 3 per group) were collected and fixed in 4% paraformaldehyde for 24 h for subsequent histopathological analysis.

### 2.4. Histopathological Analysis

This analysis used hematoxylin and eosin (H&E) staining on formalin-fixed tissue samples, which were dehydrated with ethanol, cleared with xylene, and embedded in paraffin. Sections of 5 μm were cut with a Leica RM2016 microtome (Leica Microsystems GmbH, Wetzlar, Germany). After deparaffinization and rehydration, sections were stained with Harris hematoxylin for nuclei and eosin for cytoplasm. They were then dehydrated, cleared, mounted with neutral balsam, and examined under a Nikon Eclipse E100 microscope (Nikon, Tokyo, Japan), following [13] protocol.

### 2.5. RT-qPCR

Total RNA was extracted from ch-ECs using Trizol reagent (Tiangen, Beijing, China) and reverse transcribed to cDNA using PrimeScript RT Master Mix (TaKaRa, Beijing, China). RT-qPCR was performed using SYBR Green (TaKaRa, Beijing, China). Relative gene expression was quantified by qRT-PCR, and the data were analyzed using the comparative Ct method (2^^(−ΔΔCt)^), with 18s-rRNA serving as the internal reference gene for normalization. Differences between the two groups were assessed using Student’s *t*-test, and a *p* value < 0.05 was considered statistically significant. Primer sequences are listed in Table S1.

### 2.6. Multi-Phase Transcriptomic Strategy and Experimental Design

**Figure 1:**
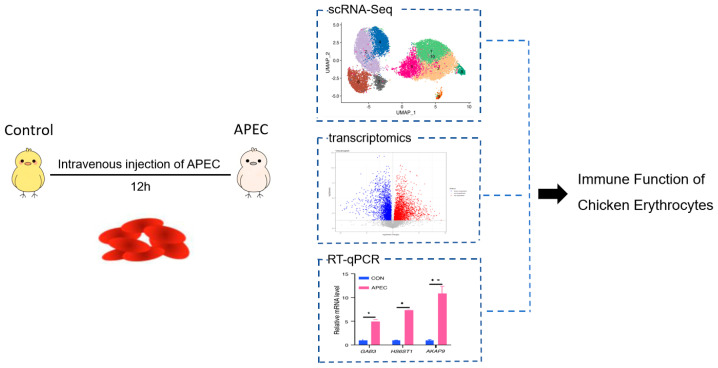
Multi-phase transcriptomics analysis of chick erythroid cells’ response to APEC infection.

This study employed a sequential three-phase transcriptomic strategy (Figure 1), informed by resource considerations, which encompassed the stages of exploration, validation, and precise quantification.

#### 2.6.1. scRNA-Seq Exploration

To facilitate an unbiased investigation of cellular heterogeneity and the generation of initial hypotheses, one chick from each of the CON and APEC-infected groups was randomly selected for scRNA-seq using ch-ECs isolated from peripheral blood. This approach aimed to delineate cell subpopulations and identify key candidate genes.

#### 2.6.2. Bulk RNA-Seq Validation

To overcome the small sample size limitation of scRNA-seq and validate the initial scRNA-seq findings, bulk RNA sequencing was conducted on ch-ECs isolated from a separate cohort of chicks (*n* = 3 per group). These chicks were reared under identical age and treatment conditions; however, they were housed and processed independently at distinct time points.

#### 2.6.3. RT-qPCR Quantification Phase

For further confirmation of key genes, RNA extracted from ch-ECs of another completely independent sample set (*n* = 3 per group) was used for RT-qPCR analysis.

This comprehensive design adheres to a rigorous research framework encompassing discovery, validation, and quantification, thereby systematically elucidating the mechanisms of immune response in ch-ECs during infection.

### 2.7. Single-Cell RNA Sequencing

#### 2.7.1. Single-Cell Transcriptome Library Prep, Sequencing, and Data Analysis

Single-cell GEMs were prepared with the Chromium Next GEM Single Cell 3’ Kit v3.1 (10 × Genomics), and cDNA libraries were constructed, according to the manufacturer’s protocol. Libraries were sequenced on an Illumina NovaSeq 6000 (Oebiotech, Shanghai, China). FASTQ files were processed with Cell Ranger v5.0.0 to obtain UMI count matrices, which were analyzed in Seurat v4.0.0. Cells with <200 genes, <1000 UMIs, log10 (GenesPerUMI) < 0.7, or >10% mitochondrial UMIs were removed; doublets were screened using DoubletFinder v2.0.2. Data were LogNormalized (scale factor 10,000); highly variable genes were selected as described by [14] via FindVariableGenes, followed by PCA and UMAP for dimensionality reduction. Cluster markers were identified with FindAllMarkers and visualized with VlnPlot/FeaturePlot; cell-type annotation was performed using Spearman correlation against a public single-cell reference with SingleR v1.4.1.

#### 2.7.2. Differential Expression, Functional Enrichment, Pseudotime, and Regulatory Network Analysis

Differentially Expressed Gene and Functional Enrichment Analysis. Differential expression analysis between ch-ECs and ch-EC subpopulations from APEC-infected and CON groups was performed using the FindMarkers function in Seurat. The presto package was used for differential testing, applying thresholds of *p* value < 0.05 and absolute fold change > 1.5. Gene Ontology (GO) and Kyoto Encyclopedia of Genes and Genomes (KEGG) enrichment analyses were conducted on the identified differentially expressed genes (DEGs) using a hypergeometric distribution test.

Pseudotime Trajectory Analysis. The Monocle 3 software package was used to analyze ch-ECs from both CON and APEC-infected samples. Genes with large differential expression were used for dimensionality reduction, after which a minimum spanning tree (MST) was constructed. The longest paths within the MST were identified to represent differentiation trajectories of cells with similar transcriptional profiles. Significant genes, grouped into discrete modules, were visualized using the plot_pseudotime_heatmap function.

SCENIC Analysis. The regulatory network of ch-ECs was analyzed using the SCENIC method. GENIE3 was used to infer co-expression relationships between transcription factors (TFs) and candidate target genes. Cis-regulatory motifs were analyzed for each co-expression module using RcisTarget. TF-motif enrichment analysis was performed to identify direct targets, with each processed TF and its potential direct target genes defined as a regulon. Regulon activity in individual cells was scored using the AUCell algorithm, and stable cellular states were determined based on regulon activity for further exploration.

### 2.8. Bulk RNA-Seq

Total RNA was extracted from ch-ECs (CON and APEC-infected groups; *n* = 3 each) using TRIzol reagent. After assessing the RNA quality (including integrity analysis via Agilent 2100 Bioanalyzer, Agilent Technologies, Santa Clara, CA, USA) and quantity, cDNA libraries were prepared according to the manufacturer’s instructions for the RNA-seq library preparation kit (VAHTS Universal V5 RNA-seq Library Prep Kit, Vazyme Biotech, Nanjing, China). The resulting libraries were sequenced on the Illumina NovaSeq 6000 platform (Illumina, San Diego, CA, USA) to generate 150-bp paired-end reads. Raw sequencing reads were quality-controlled using FASTQ v2.20 to obtain clean data. Clean reads were aligned to the reference genome using HISAT2, and gene expression levels were quantified. Differential expression analysis was performed with DESeq2, applying thresholds of |log2FoldChange| > 1 and an adjusted *p* value < 0.05. GO and KEGG enrichment analyses were conducted on the identified DEGs to elucidate significantly enriched biological functions and pathways.

### 2.9. Statistical Analysis

Statistical significance between treatment groups was determined by Student’s *t*-test using GraphPad Prism software (v9; GraphPad Software, Inc., San Diego, CA, USA). Data obtained from at least three independent biological replicates are presented as the mean ± standard deviation (SD). A *p* value < 0.05 was considered statistically significant (*).

## 3. Results

### 3.1. Pathological Manifestations in Chick Organs and Innate Immune Responses of ch-ECs Following APEC Infection

#### 3.1.1. Pathological Manifestations in Various Organs of Chicks Infected with APEC

**Figure 2:**
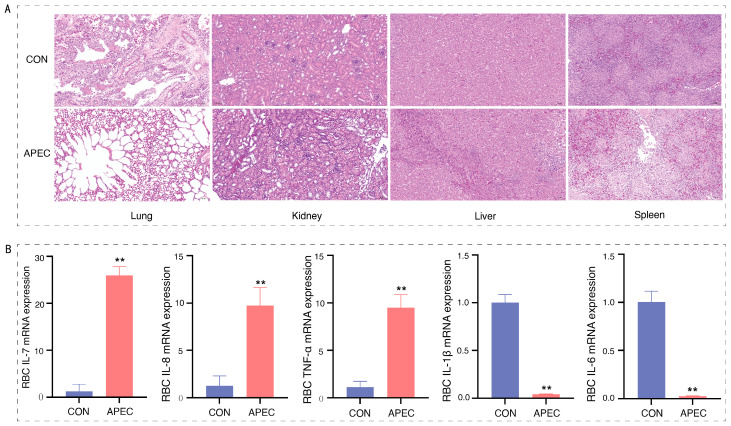
(**A**) Representative H&E-stained histopathological images of lung, kidney, liver, and spleen tissues at 200× magnification, with a scale bar of 50 μm; (**B**) alterations in cytokine gene mRNA expression in ch-ECs. Data represent mean ± SD. ** *p* < 0.01 (compared to CON group, Student’s *t*-test).

A histological examination of liver, spleen, lung, kidney, and heart tissues from both the control group and the APEC-infected group was conducted using H&E staining. The analysis revealed no significant pathological alterations in any tissues of the control group. In contrast, the APEC-infected group exhibited several pathological changes: the liver showed aggregation of inflammatory cells, along with the dissolution of cell membranes and cytoplasm at lesion sites; the lung tissue demonstrated thinning and deformation of alveolar epithelial cells and basement membranes, which appeared stretched, accompanied by alveolar vacuolation; the renal tissue exhibited unclear and deformed glomerular morphology with indistinct boundaries, along with lymphocyte infiltration surrounding the glomeruli, dissolution of renal tubular cells, and the presence of red blood cells, indicative of hemorrhage; the spleen displayed swollen white pulp and mild vacuolation (Figure 2A). The presence of these characteristic pathological lesions collectively verified the successful establishment of the APEC infection model in chicks, thereby providing a robust foundation for subsequent investigations into the host immune response.

#### 3.1.2. Expression of Innate Immune Response Genes in Chick Erythroid Cells

Cytokines serve as essential signaling molecules within the immune system, playing pivotal roles in mediating immune responses and inflammation. This investigation employed real-time quantitative PCR to assess changes in the cytokine expression levels. As illustrated in Figure 2B, post APEC infection, there was a significant upregulation in the expression levels of the cytokines *IL-7*, *IL-8*, and *TNF-α* in red blood cells (*p* < 0.01), whereas the expression levels of *IL-1β* and *IL-6* were significantly downregulated (*p* < 0.05). Consequently, monitoring alterations in the cytokine expression levels in ch-ECs from APEC-infected provides valuable insights into the immunological functions of ch-ECs.

### 3.2. Strategy for scRNA-Seq and Data Analysis of the Chick Erythroid Cells

**Figure 3:**
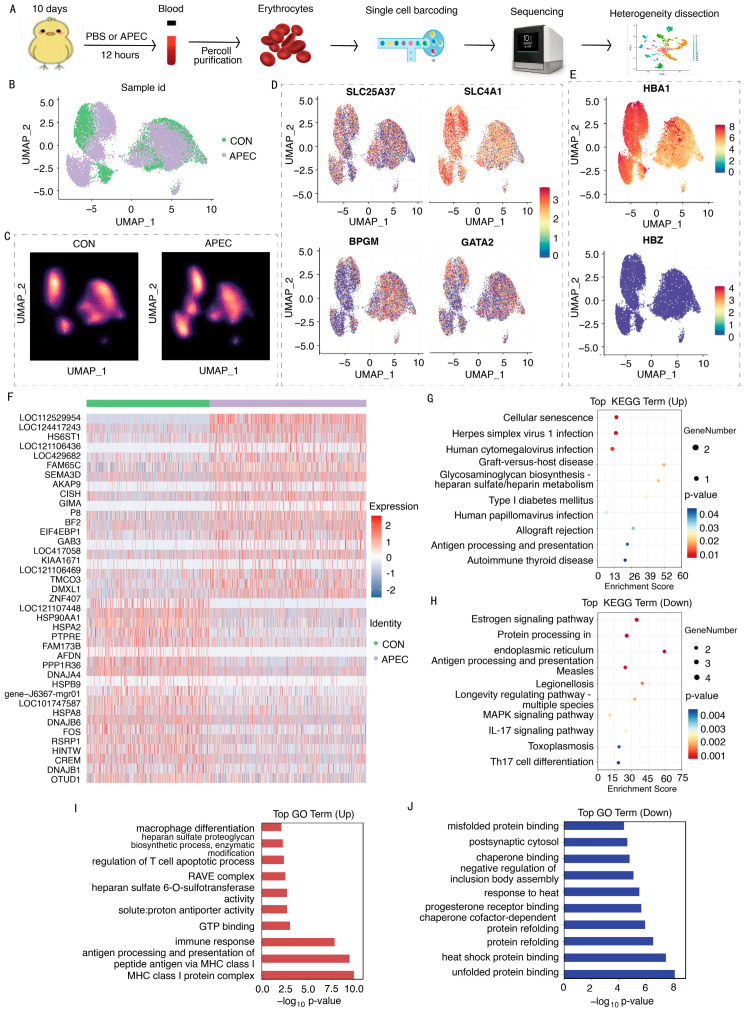
Strategy for scRNA-Seq and transcriptome analysis of ch-CEs in APEC-infected and CON samples. (**A**) Overview of scRNA sequencing analysis research. (**B**) UMAP representation of ch-ECs in APEC-infected and CON samples. (**C**) UMAP representation of ch-ECs from APEC-infected and CON individually. (**D**,**E**) UMAP visualization of typical ch-ECs marker expression. (**F**) Heatmap of DEGs in ch-ECs. (**G**) KEGG analysis of upregulated DEGs in ch-ECs. (**H**) KEGG analysis of downregulated DEGs in ch-ECs. (**I**) GO analysis of upregulated DEGs in ch-ECs. (**J**) GO analysis of downregulated DEGs in ch-ECs.

To explore the immunotranscriptome of ch-ECs, we performed scRNA-seq analysis on ch-ECs from APEC-infected and CON using 10 × Genomics (Figure 3A). Following quality filtering, scRNA-seq identified 7159 ch-ECs in the CON group, with median expression levels of 760 genes and 1507 UMIs per cell. In the APEC-infected group, 9130 ch-ECs were detected, exhibiting median counts of 798 genes and 1515 UMIs per cell (Table S2). Visualization of the APEC-infected and control samples was achieved through Principal Component Analysis and unified manifold approximation and projection (Figure 3B,C). To confirm the identity of ch-ECs, we assessed the expression of 11 canonical cell-type marker genes. Ch-ECs expressed erythroid lineage markers (*SLC25A37*, *SLC4A1*, *BPGM*, *GATA2*; Figure 3D) and exhibited the specific expression of hemoglobin gene *HBA1* [α-globin] (Figure 3E). In contrast, markers of dendritic cells, mesenchymal cells, epithelial cells, fibroblasts, macrophages, and other non-erythroid lineages were either minimally expressed or absent (Figures S1 and S2). These results demonstrate the successful isolation of ECs from chicks and the acquisition of high-quality scRNA-seq data, which underwent stringent quality control for downstream research.

A comparative transcriptomic analysis of ch-ECs from APEC-infected and CON groups was conducted. Differential expression analysis identified 38 significantly regulated genes, comprising 19 upregulated and 19 downregulated genes (Figure 3F). Among the upregulated genes, several key immune and signaling-related genes were prominent, including the following: *HS6ST1*, encoding a type II transmembrane protein involved in modulating extracellular signaling molecule activity and distribution; *AKAP9*, a scaffolding protein gene critical for signal transduction regulation; *GAB3*, associated with macrophage differentiation; *SEMA3D*, encoding a signaling protein; *CISH*, which encodes cytokine-induced STAT inhibitory factor (CIS); *EIF4EBP1*, involved in translational regulation via binding to eukaryotic initiation factor 4E (eIF4E); and *BF2*, a gene implicated in antigen presentation and processing. Conversely, notable downregulation was observed in genes including the following: *AFDN*, an adhesion junction formation factor; *FOS*, a key transcription factor; heat shock protein genes such as *HSP90AA1*, *HSPA2*, *HSPA8*, and *HSPB9*; and chaperone protein genes such as *DNAJA4* and *DNAJB1*. KEGG pathway analysis revealed the significant enrichment of viral infection-related pathways and glycosaminoglycan biosynthesis, particularly heparan sulfate/heparin metabolism (Figure 3G). In contrast, the antigen processing and presentation pathway, as well as the MAPK signaling pathway, were markedly downregulated (Figure 3H). GO enrichment further highlighted upregulated biological processes, including MHC class I protein complex assembly, antigen processing and presentation, immune response regulation, GTP binding, and acetylheparan sulfate 6-O- sulfotransferase activity (Figure 3I). Conversely, the heat shock response process, including unfolded protein binding, heat shock protein binding, protein refolding, and thermal stress response, was significantly downregulated (Figure 3J). Collectively, the upregulation of immune signaling and antiviral pathways, along with the suppression of heat shock responses, indicates that ch-ECs mount an active immune defense against APEC infection.

### 3.3. Immune Heterogeneity of Erythroid Cells

**Figure 4:**
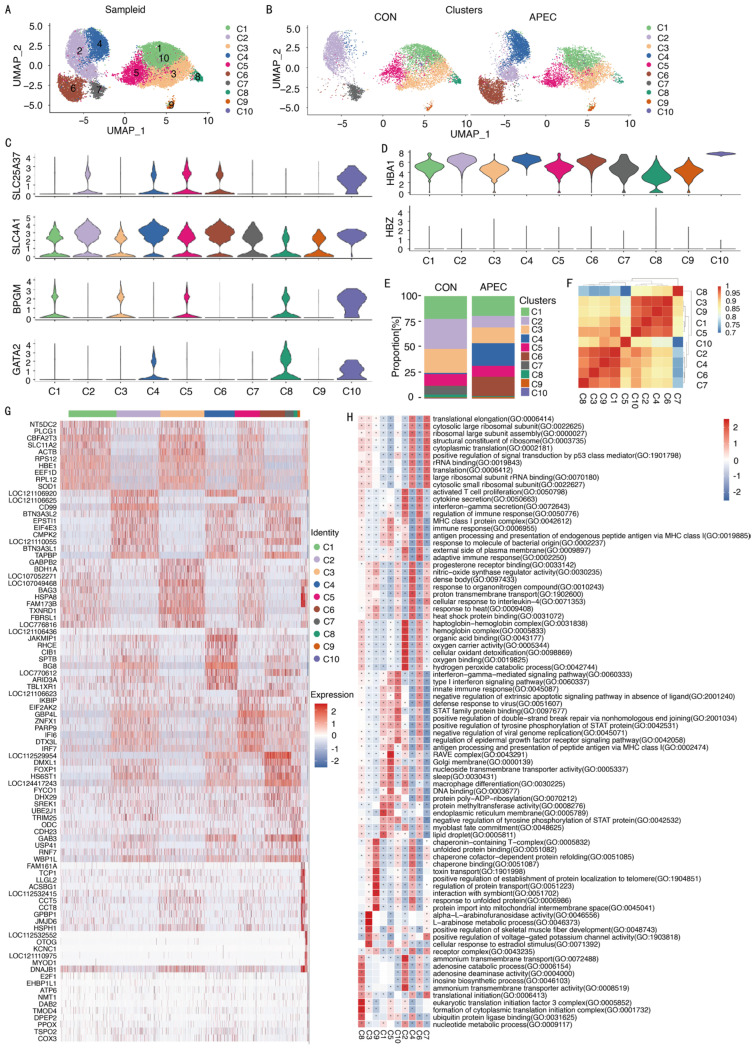
ScRNA-seq reveals the heterogeneity of ch-ECs. (**A**) UMAP of ch-EC subpopulations. Different colors represent different cell subpopulations. (**B**) UMAP of ch-EC subpopulations in APEC-infected and CON samples. (**C**,**D**) Violin plots of the expression levels of typical EC markers in ch-EC subpopulations. (**E**) Bar chart shows the proportion of ch-EC subpopulations in APEC-infected and CON samples. (**F**) Heatmap shows the correlation of ch-EC subpopulations. (**G**) Heatmap shows the top 10 DEGs of ch-EC subpopulations. (**H**) Heatmap of GO enrichment analysis of DEGs. The asterisk (*) indicates significantly enriched pathways (FDR-corrected *p* < 0.05).

Unsupervised t-SNE analysis revealed 10 distinct subpopulations (designated C1–C10) within ch-ECs (Figure 4A and Table S3). Figure 4B illustrates the ten distinct subpopulations within ch-ECs from APEC-infected and CON samples. Ch-EC subpopulations were positive for the expression of the erythroid lineage markers such as *SLC25A37*, *SLC4A1*, *BPGM*, and *GATA2* (Figure 4C), as well as hemoglobin genes such as HBA1 and HBZ (Figure 4D). Notably, subpopulations C2, C4, C6, and C7 displayed significant compositional differences between APEC-infected and CON samples (Figure 4E and Table S4).

To investigate the relationships among ch-EC subpopulations, we computed Pearson correlation coefficients based on their average gene expression profiles (Figure 4F). Our analysis revealed strong correlations among subpopulations C1, C3, C5, and C9, as well as among C2, C4, C6, C7, and C10. In contrast, the C8 subpopulation displayed minimal correlation with the other nine subpopulations (r < 0.2), suggesting a distinct transcriptional profile.

The immune heterogeneity of chEC subpopulations (Figure 4G and Table S5) was shown as follows: The C1 subpopulation highly expressed the genes *HBE1* that encodes hemoglobin, SOD that encodes superoxide dismutase, *SLC11A2* and *NT5DC2*, which are linked to metal ion transport, along with *ACTB* and *PLCG1*, which respond to bacterial infection. Additionally, high expression of transcriptional regulators *CBFA2T3* and *EEF1D*, ribosomal components *RPS12* and *RPL12*, and the nucleotidase gene *NT5DC2* indicated an actively developing state in this population. The C2 subpopulation showed elevated expression of adaptive immunity genes (*CD99*, *TAPBP*, *BTN3A3L1*, *BTN3A3L2*), where *BTN3A3L1* and *BTN3A3L2* regulate interferon-mediated signaling. Additionally, *EPSTI1* related to Nuclear Factor Kappa-light-chain-enhancer of Activated B Cells (NF-κB) pathway and *CMPK2* related to LPS response were upregulated. The C3 subpopulation highly expressed *FBRSL1*, involved in protein assembly, and *TXNRD1*, associated with redox regulation and apoptosis. The C4 subpopulation showed high expression of *BG8*, related to adaptive immunity, along with *SPTB* and *TBL1XR1*, which participate in the MAPK cascade. The C5 subpopulation exhibited elevated levels of *IRF7*, involved in the TLR signaling pathway; *DTX3L*, linked to the Notch signaling pathway; *GBP4L*, participating in the NLR signaling pathway; *PARP9*, responsive to gamma interferon; and *IFI6*, encoding interferon alpha-inducible protein 6, indicating roles in immune regulation and signaling pathways. The C6 subpopulation displayed high expression of *HS6ST1*, related to heparan sulfate proteoglycan biosynthesis; *DMXL1*, associated with intracellular signal transduction and membrane trafficking; and *FYCO1*, involved in lysosome and vesicle translocation. The C7 subpopulation showed upregulated *GAB3*, implicated in macrophage differentiation; *ODC*, a metabolism-related gene; *UBE2J1*, *USP41*, and *RNF7*, involved in the ubiquitination process; *TRIM25*, regulating the NF-κB/RIG-like receptor signaling pathway; and *CDH23*, an adhesion-related gene. The C9 subpopulation showed high expression of heat shock genes *HSPH1* and *DNAJB1*. Finally, the C10 subpopulation upregulated *ATP6*, *COX3*, and *PPOX*, genes associated with oxidative biological processes. In summary, we successfully delineated heterogeneous cell types at single-cell resolution, characterizing their immune-and cell cycle-related gene signatures. These findings provide a molecular framework for understanding the immunobiology of ch-ECs.

To further investigate the immune functions of ch-ECs, we performed GSVA analysis on the subpopulation’s marker genes. The GO enrichment analysis revealed the following (Figure 4H): C2 exhibited enrichment in immune response regulation, cytokine secretion, response to bacterial-derived molecules, MHC class I antigen presentation, and the adaptive immune response. C3 displayed significant associations with the regulation of nitric oxide synthase activity, response to organic nitrogen compounds, heat shock response, and IL-4 response. C4 showed enrichment in hemoglobin complex formation, oxygen carrier activity, cellular oxidative detoxification, oxidative binding, and hydrogen peroxide metabolism. C5 showed enrichment in interferon signaling, regulation of STAT protein tyrosine phosphorylation, modulation of epidermal growth factor receptor signaling, antiviral defense response, and immune response regulation. C6 demonstrated notable involvement in MHC class I antigen presentation and significant immune response modulation. C7 exhibited enrichment in STAT protein tyrosine phosphorylation, MHC class I—mediated antigen presentation, and antiviral defense mechanisms. C9 showed enrichment in heat shock protein activity and unfolded protein binding. C10 showed enrichment in ubiquitin protein ligase binding processes. KEGG pathway analysis (Figure S3) identified key signaling pathways: The Rap1 signaling pathway was significantly enriched in C1, C3, and C9. The NLR signaling pathway was prominent in C5. The RIG-I-like receptor signaling pathway was notably activated in C7.

### 3.4. Transition Trajectories and Transcriptional Regulatory Network of Erythroid Cells

**Figure 5:**
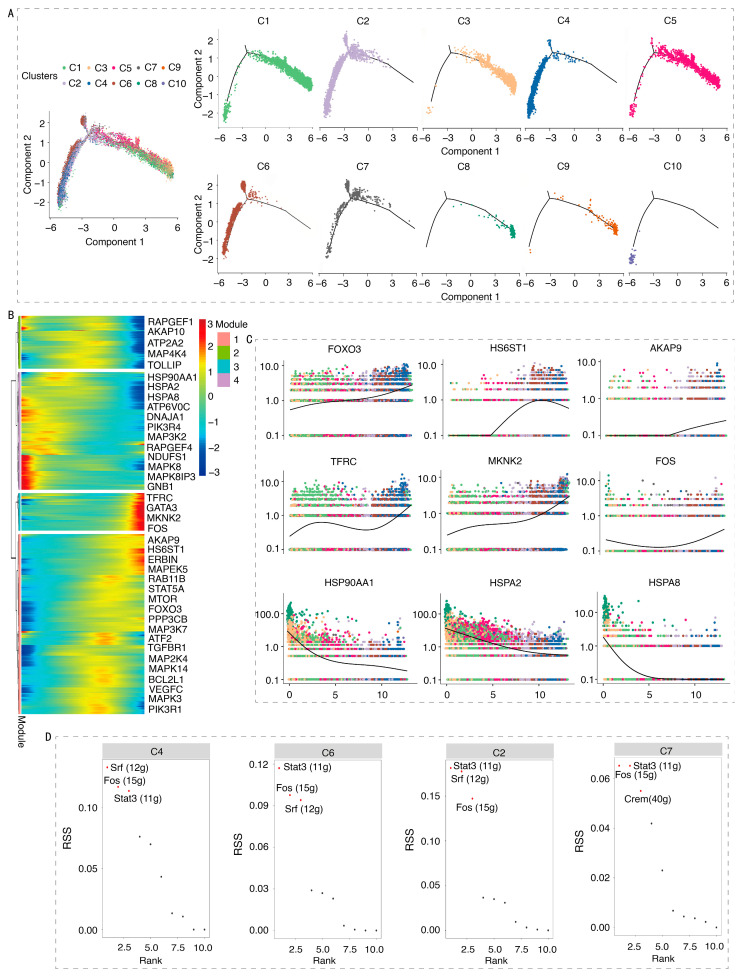
Pseudotemporal dynamics and SCENIC analysis of ch-ECs from APEC- infected and CON samples. (**A**) Pseudotime analysis based on ch-EC subpopulations. Axis from right to left indicates ch-ECs’ progression from the early to the late stage, with individual cell clusters represented by distinct colors. (**B**) Heatmap shows gene expression changes along pseudotime. Cells on the right correspond to the naive cell state. (**C**) Pseudotime-ordered single-cell expression trajectories of genes. The horizontal axis, oriented from left to right, represents the temporal progression from early to late stages, whereas the vertical axis indicates the gene expression levels. Distinct cell populations are depicted by colored dots, and the curve demonstrates the trend in gene expression. (**D**) The ranked plot shows ch-EC-specific regulons in descending activity order based on SCENIC analysis. The horizontal axis denotes the ranking, while the vertical axis represents the RSS score. Regulons with elevated RSS scores are more likely to be specific to the corresponding cell cluster.

Pseudotime trajectory analysis revealed the development status of ch-ECs derived from APEC-infected and CON samples (Figure S5A,B). The results revealed a clear stratification of developmental states (Figure 5A): subpopulations C1, C3, C5, and C9 were predominantly positioned along the early developmental trajectory, whereas C2, C4, C6, C7, and C10 exhibited transcriptional profiles consistent with a more mature state. Notably, the C8 subpopulation exhibited characteristics of naive cells. This study analyzes the developmental stages of ch-EC subpopulations, providing a reference for further research on their immune functions. Pseudotime analysis comparing APEC-infected and CON samples revealed significant immune-related molecular changes in ch-ECs. Four distinct transcriptional phases were identified, along with 62 DEGs associated with immune regulation (Figure 5B and Table S6). The dynamic expression of the key immune modulators is illustrated in Figure 5C. Phase I showed significant upregulation of the transcription factor *FOXO3* along with immune-related genes *AKAP9* and *HS6ST1*. Phase III showed predominant later-phase upregulation of *TFRC* which encodes transferrin receptor protein, *MKNK2* which encodes serine/threonine kinase, and the transcription factor *FOS*. In contrast, phase IV exhibited marked downregulation of heat shock genes including *HSP90AA1*, *HSPA2*, and *HSPA8*. The pseudotime trajectory results demonstrate dynamic transcriptional reprogramming in ch-ECs during APEC infection, suggesting their crucial role in immune response modulation through coordinated regulation of key immune-related genes.

To elucidate the key TFs involved in ch-ECs response to APEC infection, SCENIC analysis was performed on ch-EC subpopulations from APEC-infected and CON samples. Figure S4C characterizes the representative transcription factors of erythrocyte subsets, presenting their activity dynamics across each subset. This analysis predicted TF activity changes across ch-EC subpopulations, identifying 11 significantly altered regulons (Figure S4D). Notably, the regulons of *Mxi1* (33 g), *E2f1* (15 g), *Foxk2* (19 g), *Fos* (15 g), and *Srf* (12 g) exhibited the highest activity (Figure S4E). Subpopulation-specific TF analysis revealed *Fos* (15 g), *Srf* (12 g), and *Stat3* (11 g) as dominant regulators in distinct ch-EC populations (Figure 5D). Regulatory network assessment demonstrated strong associations between specific regulons: *Fos* (15 g) correlated highly with *Jund* (28 g) and *Mxi1* (33 g), *Srf* (12 g) showed significant co-regulation with *Jund* (28 g), and *Stat3* (11 g) was closely linked to *Crem* (40 g), *Yy1* (56 g), and *Foxn3* (17 g) (Figure S4F). The gene sets targeted by each TF were further characterized using SCENIC (Table S7). These findings demonstrate that ch-EC subpopulations undergo specific transcriptional reprogramming following APEC infection, primarily mediated through *Fos* (15 g), *Srf* (12 g), and *Stat3* (11 g) regulatory networks. Notably, these TFs have established roles in immune regulation, suggesting their potential importance in coordinating ch-EC immune responses during APEC challenge.

### 3.5. Transcriptome Analysis of Erythroid Cell Subpopulations Following APEC-Infection

**Figure 6:**
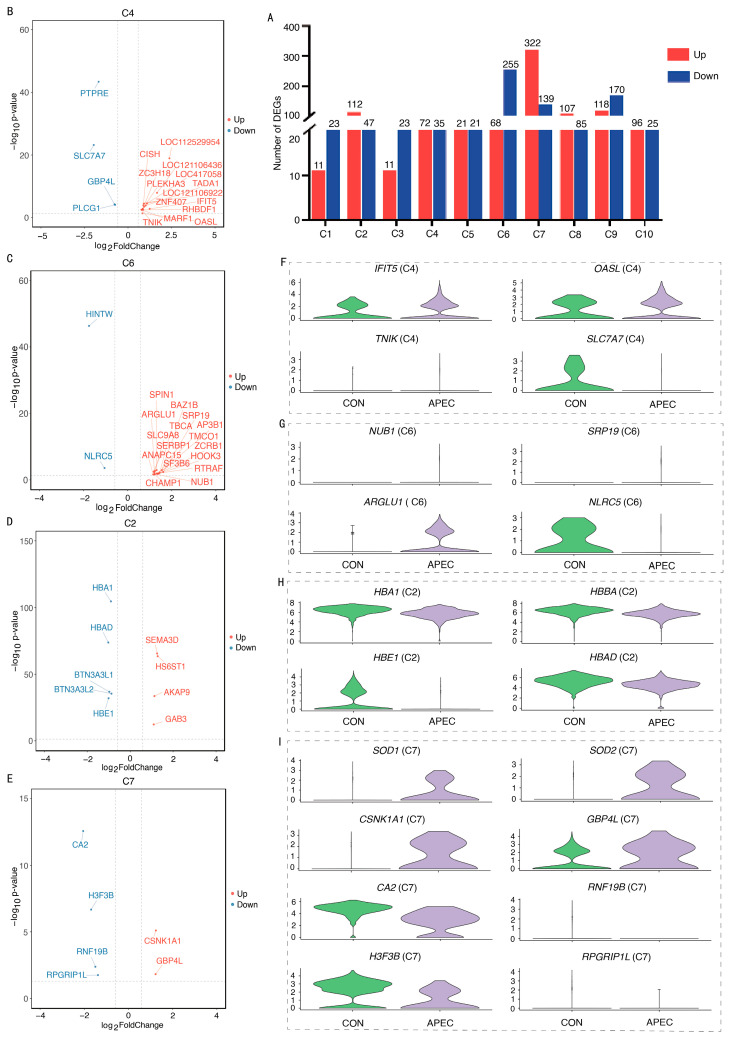
DEGs of C4, C6, C2, and C7 subpopulations between CON and APEC-infected samples. (**A**) Number of DEGs in each ch-EC subpopulation. (**B**–**E**) Volcano plots of DEGs in C4, C6, C2, and C7. (**F**–**I**) The violin plots depict differential expression of selected genes in C4, C6, C2, and C7.

To deeply understand the immune responses of different ch-EC subpopulations in chicks infected with APEC, we investigated the transcriptional profile changes in each ch-EC subpopulation and performed GO and KEGG analyses of the DEGs. Compared with other ch-EC subpopulations, the C6 and C7 exhibited the highest number of DEGs (Figure 6A and Table S8), indicating more pronounced transcriptional differences. Additionally, upon APEC infection, the abundance of C4 and C6 significantly increased, whereas that of C2 and C7 markedly decreased (Figure 4E and Table S4). Therefore, we conducted a detailed investigation into C4, C6, C2, and C7.

Transcriptome Analysis of C4 Subpopulation. C4 exhibited 107 DEGs, comprising 72 significantly upregulated and 35 significantly downregulated genes upon chick infection with APEC (Figure 6A). The analysis revealed substantial upregulation of cell cycle regulators (*IFIT5*, *TADA1*, *ZNF407*, *RHBDF1*, *ZC3H18*, *PLEKHA3*, *MARF1*) (Figure 6B and Table S8), indicating enhanced transcriptional regulation within this subset. Key upregulated immune-related genes included interferon-induced protein *IFIT5*, cytokine signaling suppressor *CISH*, antiviral effector *OASL*, and serine/threonine kinase TNIK. Additionally, four MHC class I antigen presentation-associated genes (*LOC11252-9954*, *LOC121106436*, *LOC417058*, *LOC121106922*) were significantly elevated. Conversely, notable downregulation occurred in immune modulators: TCR regulator *PTPRE*, macrophage polarization mediator *SLC7A7*, interferon-induced GTPase *GBP4L*, and metastasis-associated *PLCG1*. Figure 6F depicts the differential expression of immune-related genes between the CON group and the APEC-infected group. KEGG enrichment demonstrated significant upregulation of the MAPK signaling pathway (Figure S5A) alongside downregulation of protein processing in endoplasmic reticulum, NLR signaling, Toll/Imd signaling, Th17 cell differentiation, and Leukocyte transendothelial migration pathways (Figure S5B). GO analysis further showed enrichment in upregulated biological processes including MHC class I complex assembly, antigen presentation via MHC I, and negative regulation of viral genome replication (Figure S6A), whereas ubiquitin ligase complex, protein folding, and chaperone binding terms were significantly downregulated (Figure S6B).

Transcriptome Analysis of C6 Subpopulation. C6 exhibited a striking transcriptional shift characterized by 323 DEGs, comprising 68 significantly upregulated and 255 predominantly downregulated genes upon chick infection with APEC (Figure 6A). Substantial upregulation of cell cycle regulators (*BAZ1B*, *TBCA*, *ZCRB1*, *TMCO1*, *HOOK3*, *SPIN1*, *RTRAF*, *SLC9A8*, *CHAMP1*, *SF3B6*, *SERBP1*, *ANAPC15*) (Figure 6C and Table S8) indicated enhanced transcriptional activity. Key upregulated immune genes included viral replication suppressor *AP3B1*, interferon-induced ubiquitination modulator *NUB1*, signal recognition particle gene SRP19, and transcriptional coactivator *ARGLU1*. Conversely, critical downregulation affected apoptosis regulator *HINTW* and multifunctional NLR family member NLRC5, a modulator of MHC class I antigen presentation that suppresses NF-κB activation and type I interferon signaling. Figure 6G depicts the differential expression of immune-related genes between the CON group and APEC-infected group. KEGG analysis revealed concomitant upregulation of apoptosis, Parkinson disease, Herpes simplex virus 1 infection, and hepatocellular carcinoma pathways (Figure S5C), whereas NLR signaling, human immunodeficiency virus 1 infection, antigen processing and presentation, and ER protein processing were significantly suppressed (Figure S5D). GO enrichment further demonstrated enhanced cellular protein catabolism and anaphase-promoting complex activity alongside diminished ER-associated misfolded protein degradation (Figure S6C), unfolded protein binding, and protein folding, collectively indicating dysregulation of heat shock protein-mediated processes (Figure S6D).

Transcriptome Analysis of C2 Subpopulation. C2 displayed 159 DEGs, comprising 112 significantly upregulated and 47 downregulated transcripts upon chick infection with APEC (Figure 6A). Critical immune-related genes exhibiting upregulation included the following: *AKAP9*, which is related to scaffold protein modulating signal transduction, *HS6ST1*, which is related to type II transmembrane regulator of extracellular signaling, macrophage differentiation-associated gene *GAB3*, and semaphorin signaling gene *SEMA3D*. Conversely, pronounced downregulation targeted hemoglobin genes (*HBA1*, *HBBA*, *HBAD*, *HBE1*) and immunomodulatory butyrophilin-family genes *BTN3A3L1*/*BTN3A3L2* (Figure 6D and Table S8). Figure 6H depicts the differential expression of immune-related genes between the CON group and the APEC-infected group. KEGG analysis revealed a pro-inflammatory signature through upregulated TNF signaling and metabolic regulator mTOR signaling pathway (Figure S5E), whereas suppression of MAPK signaling and apoptosis-related pathways indicated impaired stress response coordination (Figure S5F). GO enrichment further demonstrated enhanced negative regulation of IFN-γ production and viral defense response (Figure S6E), contrasting with broad downregulation of oxidative processes: haptoglobin–hemoglobin complex formation, hemoglobin complex stability, cellular oxidative detoxification, oxygen carrier activity, oxygen binding capacity, and hydrogen peroxide catabolism (Figure S6F).

Transcriptome Analysis of C7 Subpopulation. C7 revealed 461 DEGs, of which 322 were upregulated and 139 downregulated upon chick infection with APEC (Figure 6A). Notably, the upregulated immune-related genes included *CSNK1A1*, encoding a serine/threonine-protein kinase, and *GBP4L*, an IFN-induced GTPase; conversely, the downregulated immune-related genes included *H3F3B*, which regulates immune-cell activation; *CA2*, encoding carbonic anhydrase II and involved in cellular metabolism and proliferation; *RNF19B*, a RING-type E3 ubiquitin ligase that promotes degradation of misfolded or damaged proteins; and *RPGRIP1L*, a ciliary protein regulating the Hedgehog signaling pathway (Figure 6E and Table S8). Figure 6I depicts the differential expression of immune-related genes between the CON group and APEC-infected group. KEGG pathway enrichment analysis indicated significant upregulation of oxidative phosphorylation, lysosome, autophagy, and Hedgehog signaling pathways (Figure S5G), whereas the thyroid hormone signaling and viral infection-related pathways were significantly downregulated (Figure S5H). GO enrichment further showed that cell cycle and GTPase activity-related processes were significantly upregulated (Figure S6G), whereas protein ADP-ribosylation, NAD^+^ ADP-ribosyltransferase activity, positive regulation of interferon-gamma signaling, macrophage differentiation, and intracellular steroid hormone receptor signaling pathways were significantly downregulated (Figure S6H).

### 3.6. Integrated Analysis of Bulk and Single-Cell RNA-Seq with RT-qPCR Validation

**Figure 7:**
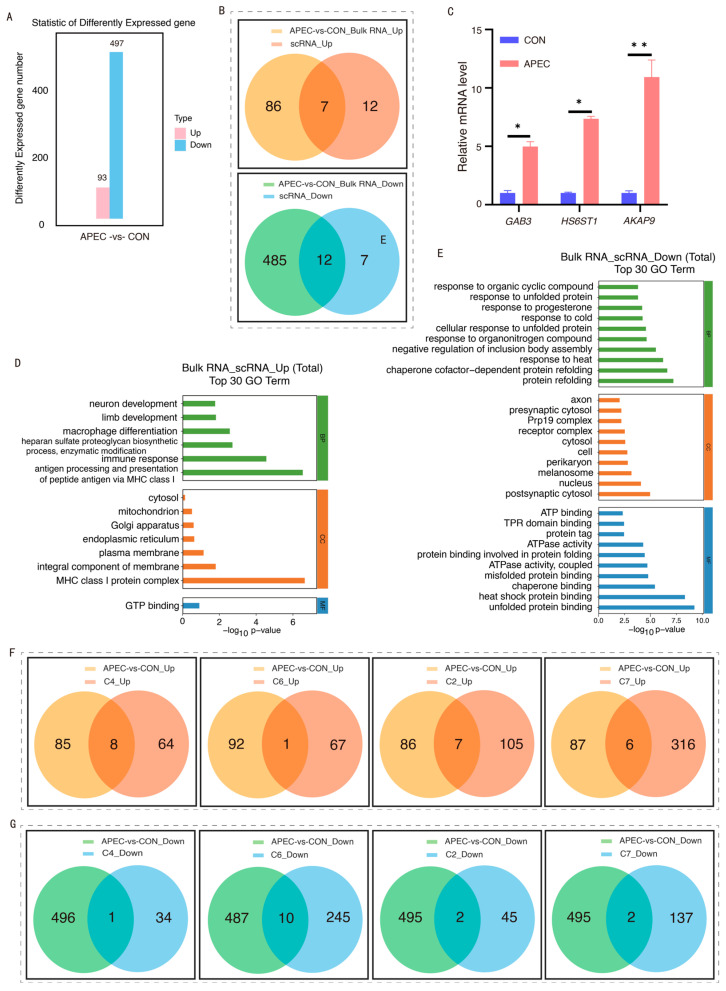
Integrated analysis of bulk and single-cell RNA-Seq with RT-qPCR validation. (**A**) Statistical chart of DEGs in ch-ECs from CON and APEC-infected samples. (**B**) An integrative analysis of DEGs derived from both scRNA-Seq and bulk RNA-Seq datasets. (**C**) Validation of genes in ch-ECs. Data represent mean ± SD. ** *p* < 0.01, * *p* < 0.05 (compared to CON group, Student’s *t*-test). (**D**) GO integrative analysis of upregulated DEGs in ch-ECs. (**E**) GO integrative analysis of downregulated DEGs in ch-ECs. (**F**) An integrative analysis of upregulated DEGs derived from both chECs subpopulations (C4, C6, C2, C7) and bulk RNA-Seq datasets. (**G**) An integrative analysis of downregulated DEGs derived from both chECs subpopulations (C4, C6, C2, C7) and bulk RNA-Seq datasets.

To validate the exploratory findings from the scRNA-seq of ch-ECs, we conducted bulk RNA-seq on ch-ECs from both the CON and APEC-infected groups. A pairwise comparison of ch-ECs between the CON and APEC-infected groups identified 17,071 co-expressed genes. Utilizing the criteria of an absolute fold change higher than 2 and a *p*-value less than 0.05, we identified 590 DEGs (Table S9). Of these, 93 genes were upregulated, while 497 genes were downregulated, with the latter representing the majority (Figure 7A).

An integrative analysis of DEGs derived from both scRNA-Seq and bulk RNA-Seq datasets identified a subset of overlapping genes. Within this subset, seven genes, including *AKAP9*, *GAB3*, *HS6ST1*, and *GIMAP8*, consistently exhibited elevated expression levels. Conversely, twelve genes, such as *FOS*, *AFDN*, *HSPA8*, *HSPA2*, *HSP90AA1*, *HSPB9*, *DNAJB1*, *DNAJB6*, and *DNAJA4*, demonstrated reduced expression levels (Figure 7B). To corroborate these observations, RT-qPCR was employed to assess the mRNA expression of selected DEGs in ch-ECs. The RT-qPCR results aligned with the sequencing data, showing significant upregulation of *AKAP9*, *HS6ST1*, and *GAB3* in ch-ECs derived from APEC-infected (Figure 7C).

We conducted GO (Figure S7A,B) and KEGG enrichment analyses (Figure S7C,D) on the DEGs identified from the bulk RNA sequencing data. These findings were then integrated with the GO and KEGG enrichment results obtained from single-cell DEGs. The GO enrichment analysis demonstrated that the upregulated biological processes were significantly associated with terms such as immune response, MHC class I protein complex, and antigen processing and presentation via MHC class I (Figure 7D). Conversely, the downregulated biological processes included the response to heat, chaperone cofactor-dependent protein refolding, protein refolding, heat shock protein binding, and unfolded protein binding (Figure 7E). The KEGG pathway analysis revealed that the upregulated pathways encompassed Glycosaminoglycan biosynthesis, specifically heparan sulfate/heparin (Figure S8A), whereas the downregulated pathways included the MAPK signaling pathway, IL-17 signaling pathway, antigen processing and presentation, and protein processing in the endoplasmic reticulum, among other significant pathways (Figure S8B).

### 3.7. Immune Function Profiling in DEGs from ch-EC Subpopulations and Bulk RNA-Seq Data

We conducted an integrative analysis of DEGs from four altered chEC subpopulations and bulk RNA-Seq data, focusing on immune-related genes. In the C4 subpopulation, eight genes, including *HS6ST1* and *GAB3*, were upregulated, while *HSP90AA1* was downregulated. The C6 subpopulation had ten downregulated genes, such as *HSPA8* and *HSP90AA1*. The C2 subpopulation showed upregulation of seven genes, including *HS6ST1*, *GAB3*, and *AKAP9*, and downregulation of *AFDN* and *FOS*.

**Figure 8:**
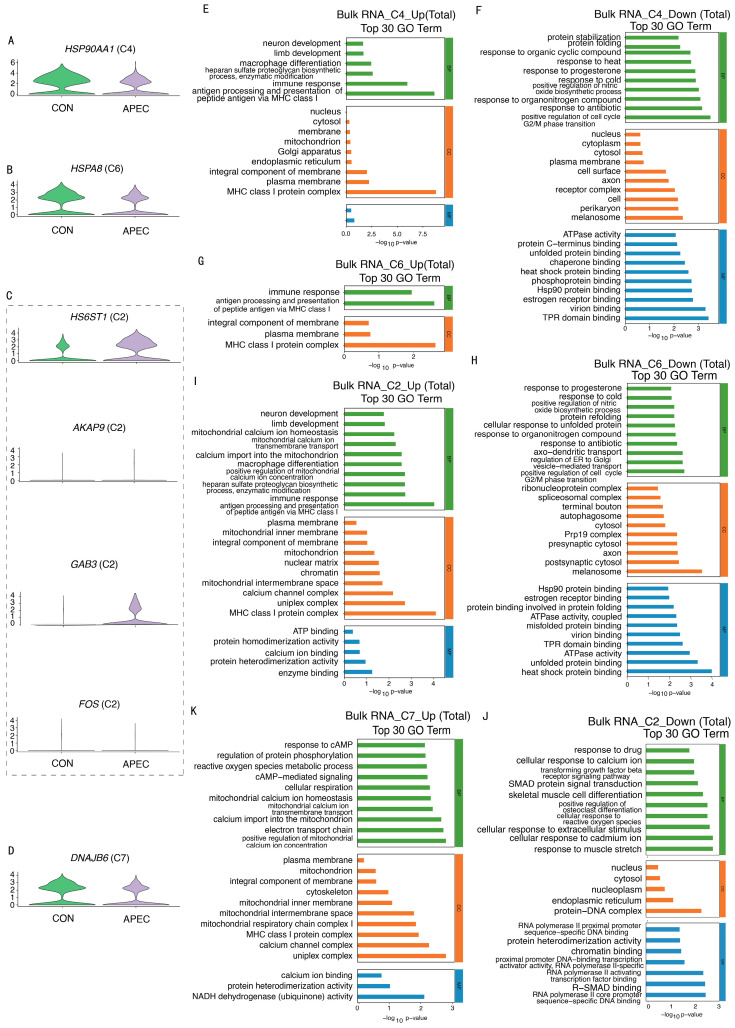
Immune function profiling at the single-cell level in chECs. (**A**–**D**) Immune genes. (**E**,**G**,**I**,**K**) Upregulated biological processes. (**F**,**H**,**J**) Downregulated biological processes.

The C7 subpopulation had six upregulated genes, including *TBCEL*, *MICU2*, and *NDUFS4*, and two downregulated genes, *DNAJB6* and *CCDC167* (Figure 7F,G). Analysis of the DEGs from four altered subpopulations, single-cell, and bulk RNA-Seq showed consistent upregulation of immune-related genes *HS6ST1*, *GAB3*, and *AKAP9* and downregulation of *FOS*, *HSPA8*, *DNAJB6*, and *HSP90AA1* (Figure 8A–D). These genes could serve as key immunological markers for the immune status of ch-ECs during APEC infection in chicks.

We performed an integrated analysis of GO and KEGG enrichment using DEGs from bulk RNA sequencing and ch-ECs subpopulations, focusing on immune-related biological processes and pathways. GO analysis showed that upregulated processes in the C4 subpopulation were linked to the immune response, MHC class I protein complex, and antigen processing via MHC class I. Downregulated processes involved TPR domain binding, virion binding, Hsp90 protein binding, and heat response (Figure 8E,F). KEGG analysis revealed downregulated pathways such as antigen processing and presentation, IL-17 signaling, Th17 cell differentiation, and NLR signaling (Figure S8C). For the C6 subpopulation, GO analysis identified significant enrichment of upregulated processes like MHC class I protein complex and the immune response, while downregulated processes included heat shock protein binding and ATPase activity (Figure 8G,H). KEGG analysis highlighted downregulated pathways such as antigen processing, NLR signaling, IL-17 signaling, MAPK signaling, and PI3K-Akt signaling (Figure S8D). In the C2 subpopulation, GO analysis revealed that upregulated processes were linked to the MHC class I protein complex, antigen processing, and immune response, while downregulated processes involved R-SMAD binding and SMAD signaling (Figure 8I,J). KEGG analysis identified downregulated pathways like cAMP, TNF, Toll-like receptor, and IL-17 signaling (Figure S8E). In the C7 subpopulation, GO analysis showed upregulated processes related to the MHC class I protein complex (Figure 8K), and KEGG analysis highlighted upregulated pathways including oxidative phosphorylation (Figure S8F). These immune processes at the single-cell level may indicate key markers for the immune status of ch-ECs during APEC infection in chicks.

## 4. Discussion

In avian species, blood volume constitutes approximately 10% of their body weight [15]. Erythroid cells represent the predominant cell type within the circulatory system. Prior research has identified novel functional genes and signaling pathways in ch-ECs at the transcriptomic level [16]. Nevertheless, investigations into the immune functions of erythroid cells remain sparse. The successful establishment of the APEC infection model was corroborated by histopathological alterations in key organs (Figure 2A), thereby providing a foundation for further investigation into the immune functions of ch-ECs. Cytokine analysis demonstrated that APEC infection specifically modulates the expression patterns of inflammation-related cytokines in ch-ECs. Notably, the significant upregulation of the potent neutrophil chemokine *IL-7* and the core inflammatory regulator *TNF-α* strongly implies an active role for ch-ECs in host innate immunity against infection. This finding aligns with conclusions drawn in previous research [17,18]. Our findings further substantiate the previously underrecognized non-classical role of ch-ECs in immune responses. Through our comprehensive analysis of single-cell and bulk RNA sequencing data, we elucidated the transcriptional regulatory mechanisms governing ch-ECs following infection with APEC. Additionally, preliminary scRNA-seq evidence suggested the presence of potential cellular heterogeneity. Although this technology has been utilized in recent years to explore the heterogeneity of human erythroid cells, there have been no published studies on the single-cell transcriptome of ch-ECs. This study presents pioneering single-cell evidence elucidating the immunotranscriptomic regulatory mechanisms and potential heterogeneity of ch-ECs in response to APEC infection, thereby providing a valuable resource for future studies on the innate immunity of nucleated erythroid cells.

### 4.1. Immune Genes in Chick Erythroid Cells

Upon chick infection with APEC, we identified DEGs that play pivotal roles in innate immunity, thereby providing valuable insights into nucleated erythroid cells’ defense mechanisms. Following APEC challenge, we observed significant upregulation of the innate immunity-associated genes *FOS*, *AKAP9*, *HS6ST1*, *GAB3*, *TFRC*, *HSPA8*, *HSP90AA1*, and *DNAJB6* in ch-ECs. In our study, we identified FOS as a crucial transcriptional regulator in chicken erythroid cells (ch-ECs) during infection with APEC. Previous research has demonstrated that the classical FOS family member, c-Fos, forms a dimer with c-Jun, resulting in the formation of the AP-1 complex, which binds to specific promoter and enhancer motifs to mediate the conversion of extracellular signals into transcriptional responses [19]. Within the context of avian infections, FOS has been identified as a fundamental host factor that facilitates viral replication by directly promoting the transcription of viral immediate-early genes in chicken cells infected with Gallid alpha-herpesvirus 1 [20]. Our pseudotime analysis notably revealed a dynamic expression pattern of FOS during infection, characterized by an initial transient downregulation followed by a subsequent rebound. We hypothesize that the initial downregulation of FOS in ch-ECs may serve as an immunosuppressive mechanism designed to mitigate excessive inflammatory activation and tissue damage during the early stages of bacterial infection. Subsequently, the resurgence in FOS expression likely indicates a transition towards the activation of later-stage immune-effector pathways, including those associated with antigen presentation or T-cell coordination [21]. *AKAP9* encodes a scaffold protein that interacts with protein kinase A (PKA) and has been implicated in oncogenic processes [22]. *HS6ST1* catalyzes the 6-O sulfation of heparan sulfate, modulating cell signaling and adhesion [23]. *GAB3* is critical for IL-2- and IL-15-driven natural killer (NK) cell activation [24]. During the ducks’ response to lipopolysaccharide (LPS), several signaling pathways associated with the immune system are activated, notably including CD71 (transferrin receptor, TFRC) [25]. Heat-shock protein genes exhibited coordinated downregulation: HSP70 family members HSPA8 and HSP40 cochaperones DNAJB6 were significantly reduced. Recent research has elucidated that HSP70 plays a dual immunomodulatory role. It significantly enhances immune responses by activating innate immunity, as evidenced by TLR2/4 signaling, and adaptive immunity, through mechanisms such as antigen cross-presentation and Th1/Th17 differentiation [26]. The observed downregulation of HSP70 family members in this study may constitute an intrinsic protective mechanism by the organism to mitigate excessive immune activation. Ni et al. elucidated that *PtHSP40-I* exhibits binding affinity towards pathogen-associated molecular patterns, such as LPS and peptidoglycan PGN, and engages in interaction with the extracellular leucine-rich repeat domain of TLR [27]. The decreased expression of HSP70 and HSP40 is likely to synergistically contribute to the modulation of excessive immune activation.

### 4.2. Immune Signaling Pathways in Chick Erythroid Cells

This study revealed significant upregulation of the MHC I antigen presentation pathway, indicating that erythroid cells play a critical immunomodulatory role via this process during APEC infection in chicks. A low-virulence serotype of Salmonella enterica was utilized to deliver an infectious bronchitis virus immunogen through a dual-promoter vector system, effectively inducing protective immunity predominantly via the activation of chicken MHC I and MHC II. The immunized chickens demonstrated a comprehensive immune response that included humoral, mucosal, and cell-mediated immunity, facilitated by concurrent activation of MHC I and MHC II pathways, while preserving a balanced Th1/Th2 immune profile [28]. Additionally, MAPK signaling pathway and the heat shock response were significantly downregulated. It has been shown that the MAPK signaling pathway primarily participates in the production of inflammatory factors and cell activation in immune responses [29]. Research conducted by Peng et al. demonstrated that a compound extracted from a traditional Chinese medicinal formulation mitigates pro-inflammatory responses through the inhibition of the MAPK/ERK/JNK signaling pathway. This mechanism subsequently reduces lung and tracheal damage in chickens infected with *Mycoplasma gallisepticum* [30]. Higher levels of HSP were observed in chicken embryos infected with *Staphylococcus aureus* [31]. Whereas active engagement in the inflammatory response has classically been viewed as an intrinsic host strategy to combat infections, the systemic downregulation of the aforementioned signaling pathways in chicks infected with APEC suggests an immunosuppressive function. This likely represents a sophisticated regulatory mechanism to prevent hyperactivation and maintain erythrocyte immune homeostasis. Intriguingly, whereas the MAPK signaling pathway was downregulated in the C2 subpopulation, it exhibited upregulation in C4. This opposing pathway activity across cell subpopulation during infection may reflect a functional heterogeneity strategy whereby erythroid cells balance immune defense and self-preservation: specific subgroups enhance immunomodulatory capacity, whereas others mitigate autologous damage.

### 4.3. Immune Heterogeneity of Chick Erythroid Cells

Four distinct subpopulations of human NECs have been identified through single-cell analysis, which reside at different developmental stages and perform diverse functions. This study reveals that ch-ECs exhibit ten distinct ch-EC subpopulations (designated C1–C10). Pseudotime analysis indicated that the C8 subpopulation represents erythroid progenitor cells, subpopulations C1, C3, C5, and C9 represent developing erythroid cells, and subpopulations C2, C4, C6, C7, and C10 represent mature erythroid cells. Characteristic gene expression profiles revealed their unique transcriptomic signatures and corresponding immune functional characteristics. Following APEC infection in chicks, significant alterations were observed in the C2, C4, C6, and C7 subpopulations. The C4 and C6 subpopulations exhibited significantly increased cell numbers, whereas the C2 and C7 subpopulations showed marked reductions. These subpopulations displayed a substantial number of DEGs, including numerous immune-related genes, indicating their active involvement in mounting immune responses against APEC infection. The C4 subpopulation exhibited significant upregulation of immune-related genes (*IFIT5*, *OASL*, *TNIK*), along with marked downregulation of *SLC7A7*. Veronica et al. demonstrated that *IFIT5* is involved in the antiviral mechanisms of rainbow trout red blood cells [32]. Judith et al. demonstrated that a *SLC7A7* transport defect leads to impaired erythropoiesis by reducing erythropoietin [33]. The C6 subpopulation showed significant upregulation of immune-related genes (*NUB1*, *SRP19*, *ARGLU1*), while *NLRC5* was significantly downregulated, indicating its substantial involvement in immune functions. The study found that *NLRC5* is involved in regulating NF-κB activation and inflammatory cytokine production in certain cell types [34]. DEGs in the C2 subpopulation, such as *HBA1*, *HBBA*, *HBE1*, and *HBAD*, showed significantly decreased expression. Given that heme synthesis consumes large amounts of iron and that unsequestered iron can catalyze reactive oxygen species generation during sepsis or infection [35], their reduced expression may limit oxidative stress-induced damage. The C7 subpopulation exhibited significant upregulation of immune-related genes (*SOD1*, *SOD2*, *CSNK1A1*, *GBP4L*) and significant downregulation of *CA2*, *RNF19B*, *H3F3B*, and *RPGRIP1L*, indicating its notable role in immune responses. Finally, superoxide dismutase genes *SOD1* and *SOD2* were significantly upregulated; their increased expression likely mitigates ROS-mediated erythrocyte injury and maintains intracellular redox balance. Research has demonstrated that superoxide dismutase (SOD) inhibits the generation of free radicals by neutralizing reactive precursors and deactivating pro-oxidant catalysts [36]. Additionally, the C1, C5, and C9 subpopulations appear to mediate certain immune functions through the downregulation of heat stress-related gene expression. These insights provide valuable leads for the subsequent interpretation of erythrocyte immune functions. Future research efforts will concentrate on expanding the single-cell cohort to validate these findings and undertaking additional mechanistic studies.

We acknowledge that a principal limitation of this study is the relatively modest sample size in the ScRNA-seq component. This design decision was guided by considerations of resource allocation for this initial exploratory-phase investigation. The primary objective was to comprehensively map cellular heterogeneity and identify potential pathways, rather than to achieve substantial statistical power for population-level comparisons. While this limitation may constrain the broader applicability of the quantitative changes observed in the subpopulations, the qualitative findings such as the identification of unique subpopulations, critical immune genes, and activated pathways provide a valuable resource and a robust foundation for future research. Additionally, we conducted bulk RNA sequencing on ch-ECs with independent biological replicates. This dataset was integrated with the ScRNA-seq results for a combined analysis, which validated the key immune genes and activated pathways initially identified through single-cell profiling. This was complemented by the experimental validation of several pivotal genes using RT-qPCR with independent biological replicates. Nevertheless, we recommend that future research endeavors increase the sample size for single-cell analysis to at least three chicks per group, incorporate additional time points, and undertake functional validation to further elucidate cell type-specific responses and their underlying mechanisms. Furthermore, considering that the current study focused on hens, future research should investigate the immune function of erythrocytes in roosters and explore potential sex-based differences.

## 5. Conclusions

This study presents an inaugural preliminary single-cell transcriptomic atlas of ch-ECs, systematically revealing the heterogeneity among their cellular subpopulations and elucidating the molecular mechanisms underlying their roles in immune regulation. It elucidates the pivotal immunological functions of vascular endothelial cells in chicks following APEC infection and suggests that key immune-related genes *HS6ST1*, *GAB3*, *AKAP9*, *FOS*, *HSPA8*, *DNAJB6*, and *HSP90AA1* may serve as significant markers indicative of the immune status of erythroid cells during APEC infection in chicks. Furthermore, the upregulation of immune-related processes, such as MHC I protein complex assembly and antigen presentation, alongside the downregulation of processes like heat shock protein binding, may represent central components of the erythrocyte immune response during this infection. These findings offer a valuable theoretical framework and data resource for further investigation into the immune functions of NECs.

## Data Availability

The raw sequencing data for this study are available in the NCBI Sequence Read Archive database (SRA; https://www.ncbi.nlm.nih.gov/sra (accessed on 4 January 2026)). The scRNA-Seq and Bulk RNA-Seq data are accessible under the BioProject accession numbers PRJNA1208498 and PRJNA1301091, respectively.

## Supplementary Materials

The following supporting information can be downloaded at https://www.mdpi.com/article/10.3390/ani16020179/s1, Figure S1: Identification of characteristic genes of various cells in chick erythroid cells; Table S1: The RT-qPCR primers for verifying the accuracy of single cell transcriptome sequencing; Figure S2: Identification of characteristic genes of various cells in chick erythroid cells; Table S2: scRNA-seq data statistics; Figure S3: Heatmap of KEGG enrichment analysis of DEGs; Table S3: cell_embeddings; Figure S4: Pseudotime and SCENIC analysis of ch-ECs between APEC-infected and CON samples; Table S4: The cell counts of chick erythroid cell subpopulations and the percent of counts versus all clusters; Figure S5: KEGG enrichment analysis (up and down) for chicken erythroid cell subpopulations of C4, C6, C2, and C7; Table S5: Differentially expressed genes in chick erythroid cell subpopulations. *p* value, avg_logFC, adjusted *p* value, and the percent of cells in the clusters of interest (pct.1) versus all other clusters (pct.2) are shown; Figure S6: GO enrichment analysis (up and down) for chicken erythroid cell subpopulations of C4, C6, C2, and C7; Table S6: Differentially expressed genes of pseudotime. *p* values are shown; Figure S7: GO and KEGG enrichment analysis (up and down) for chicken erythroid cells bulk RNA-Seq DEGs; Table S7: Gene regulatory networks. HighConfAnnot, NES, and Spearman’s correlation coefficient are shown; Figure S8: KEGG enrichment integrative analysis (up and down) for DEGs identified in chicken erythroid cells using Bulk-Seq and ScRNA-Seq methodologies and in chicken erythroid cells using subpopulations (C4, C6, C2, C7) and Bulk-Seq methodologies; Table S8: Differentially expressed genes in each chick erythroid cell subpopulations of CON and APEC-infected samples. *p* value, avg_logFC, and the percent in the clusters of APEC-infected (pct.1) versus CON clusters (pct.2) are shown. Table S9: Differentially expressed genes in each chick erythroid cell between APEC-infected and CON samples. *p* value, log2FoldChange were shown.
