# Supplementary material for: Integrated Single-Cell and Bulk Transcriptomics Unveils Immune Profiles in Chick Erythroid Cells upon Avian Pathogenic Escherichia coli Infection
Source: Animals (Basel). 2026 Jan 7;16(2):179. doi: 10.3390/ani16020179 (PMC12837167; doi:10.3390/ani16020179)

Supplementary materials

Table S1. The RT-qPCR primers for verifying the accuracy of single cell transcriptome sequencing.

Table S2. scRNA-seq data statistcs.

Table S3. cell_embeddings.

Table S4. The cell counts of chick erythroid cell subpopulations, and the percent of counts versus all clusters.

Table S5. Differentially expressed genes in chick erythroid cell subpopulations. Pvalue, avg_logFC, adjusted pvalue, and the percent of cells in the clusters of interest (pct.1) versus all other clusters (pct.2) were shown.

Table S6. Differentially expressed genes of pseudo-time. Pvalue were shown.

Table S7. Gene regulatory networks. HighConfAnnot、NES、Spearman's correlation coefficient were shown.

Table S8. Differentially expressed genes in each chick erythroid cell subpopulations of CON and APEC-infected samples. Pvalue, avg_logFC, and the percent in the clusters of APEC-infected (pct.1) versus CON clusters (pct.2) were shown.

Table S9. Differentially expressed genes in each chick erythroid cell between APEC-infected and CON samples. *p* value, log_2FoldChange were shown.

Figure S1. Identification of characteristic genes of various cells in chick erythroid cells. (A) The expression of dendritic cells characteristic genes in chick erythroid cells. (B) The expression of mesenchymal cells characteristic genes in chick erythroid cells. (C) The expression of epithelial cells characteristic genes in chick erythroid cells. (D) The expression of fibroblasts characteristic genes in chick erythroid cells. (E) The expression of endocardial cells characteristic genes in chick erythroid cells.

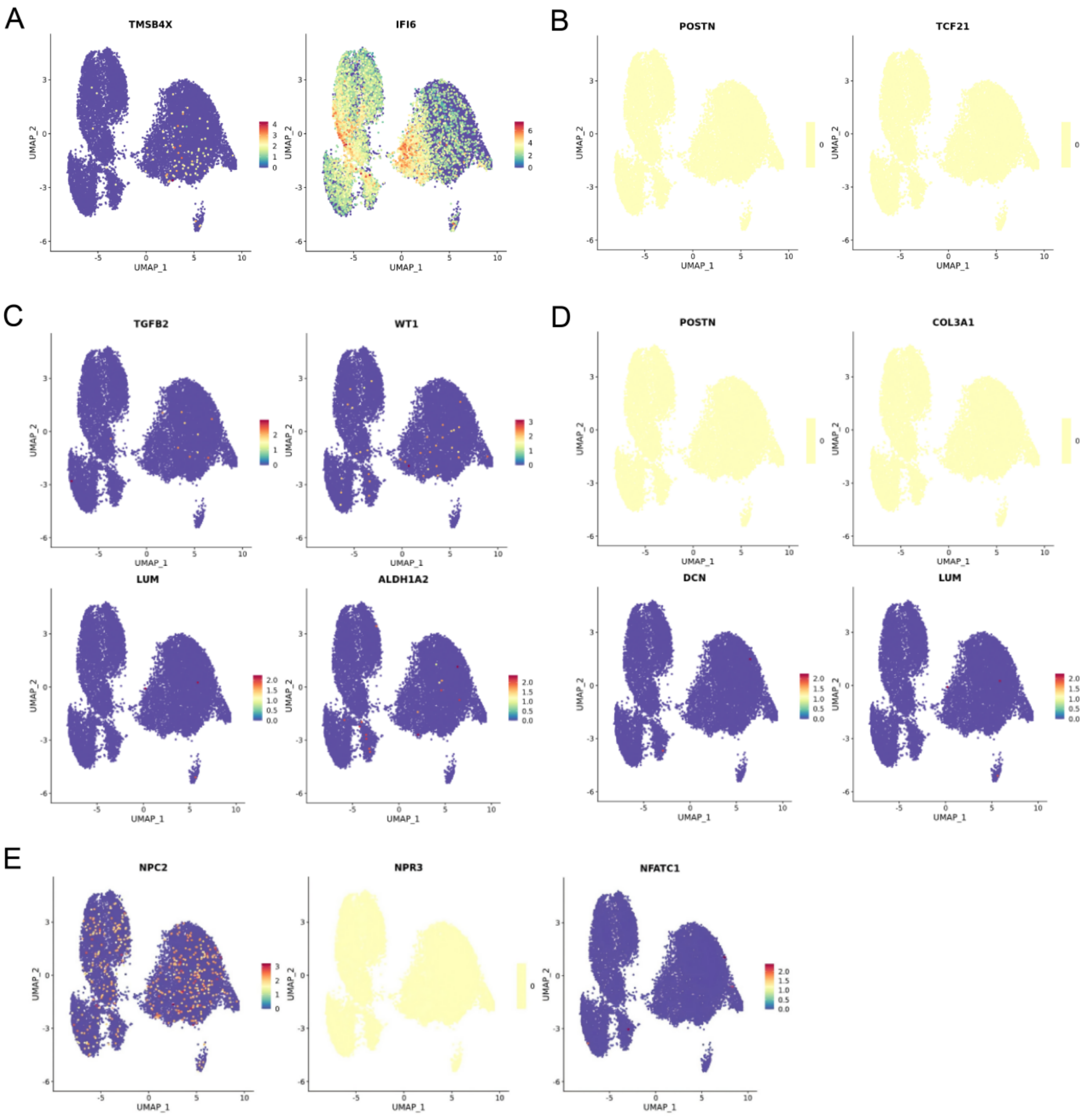

Figure S2. Identification of characteristic genes of various cells in chick erythroid cells. (A) The expression of Valve cells characteristic genes in chick erythroid cells. (B) The expression of Macrophages characteristic genes in chick erythroid cells. (C) The expression of Mural cells characteristic genes in chick erythroid cells. (D) The expression of Vascular endothelial cells characteristic genes in chick erythroid cells. (E) The expression of Immature myocardial cells characteristic genes in chick erythroid cells.

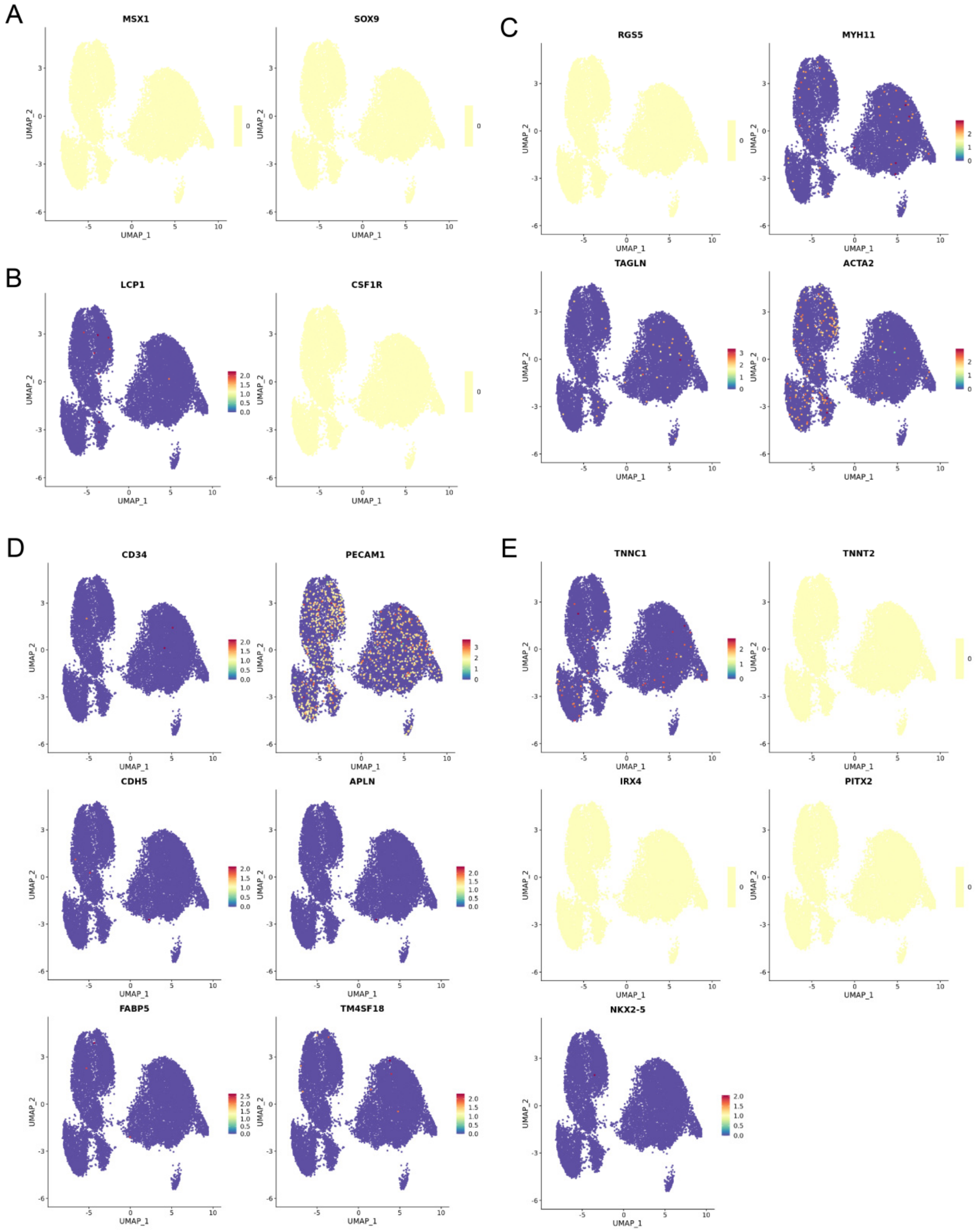

Figure S3. Heatmap of KEGG enrichment analysis of DEGs.

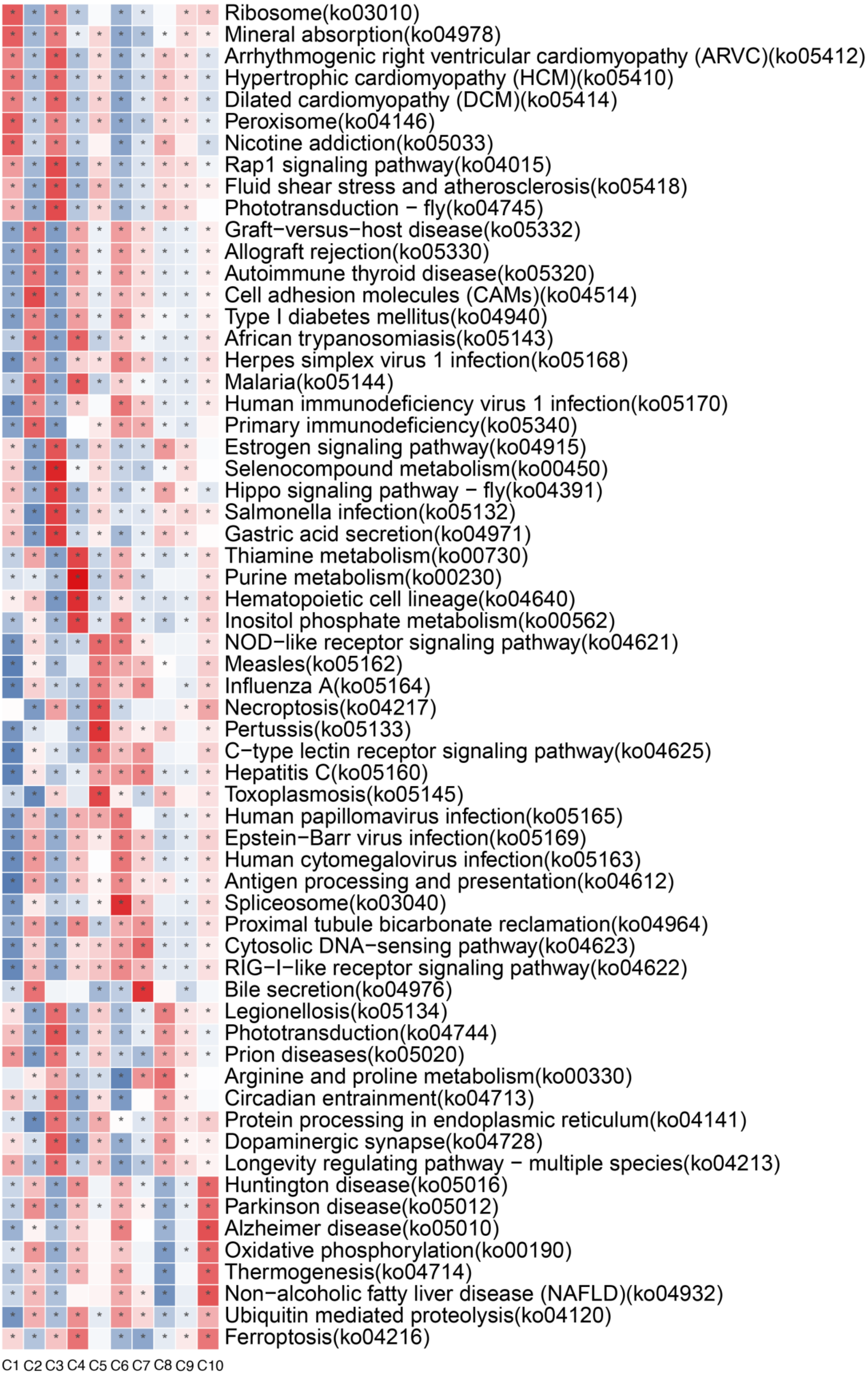

Figure S4. Pseudotime and SCENIC analysis of ch-ECs between APEC-infected and CON samples. (A-B) Pseudotime analysis of ch-ECs from APEC-infected and CON samples. (C) The heatmap shows transcription factor activity across 10 subpopulations, alongside representative factors and their target gene counts. (D) Heatmap shows the activity of TFs across ch-EC subpopulations. (E) Heatmap highlights the regulons with high activity in ch-EC subpopulations. (F) Heatmap illustrates the regulons correlation of ch-EC subpopulations.

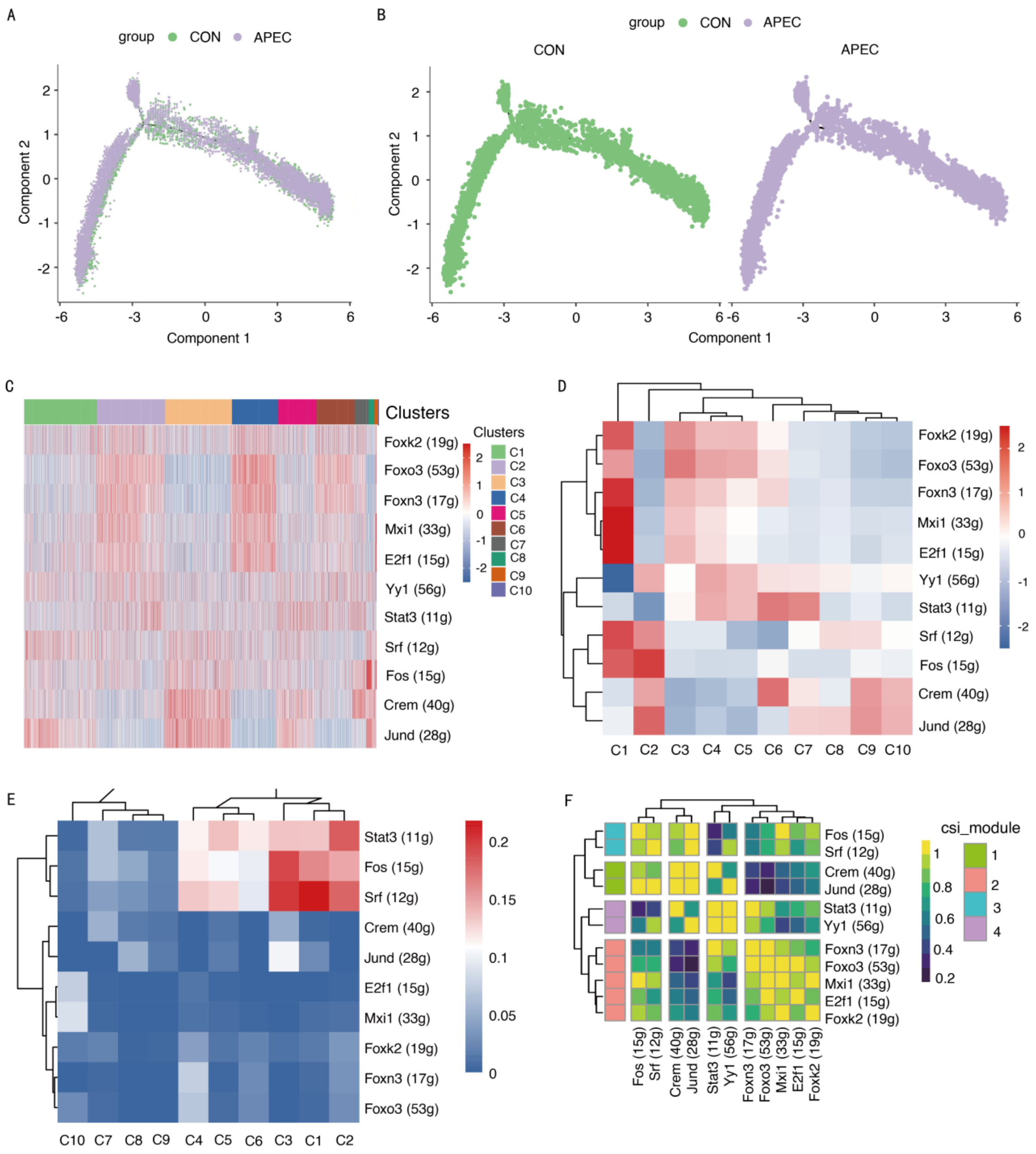

Figure S5. KEGG enrichment analysis（Up and Down）for chicken erythroid cell subpopulations of C4, C6, C2,C7.

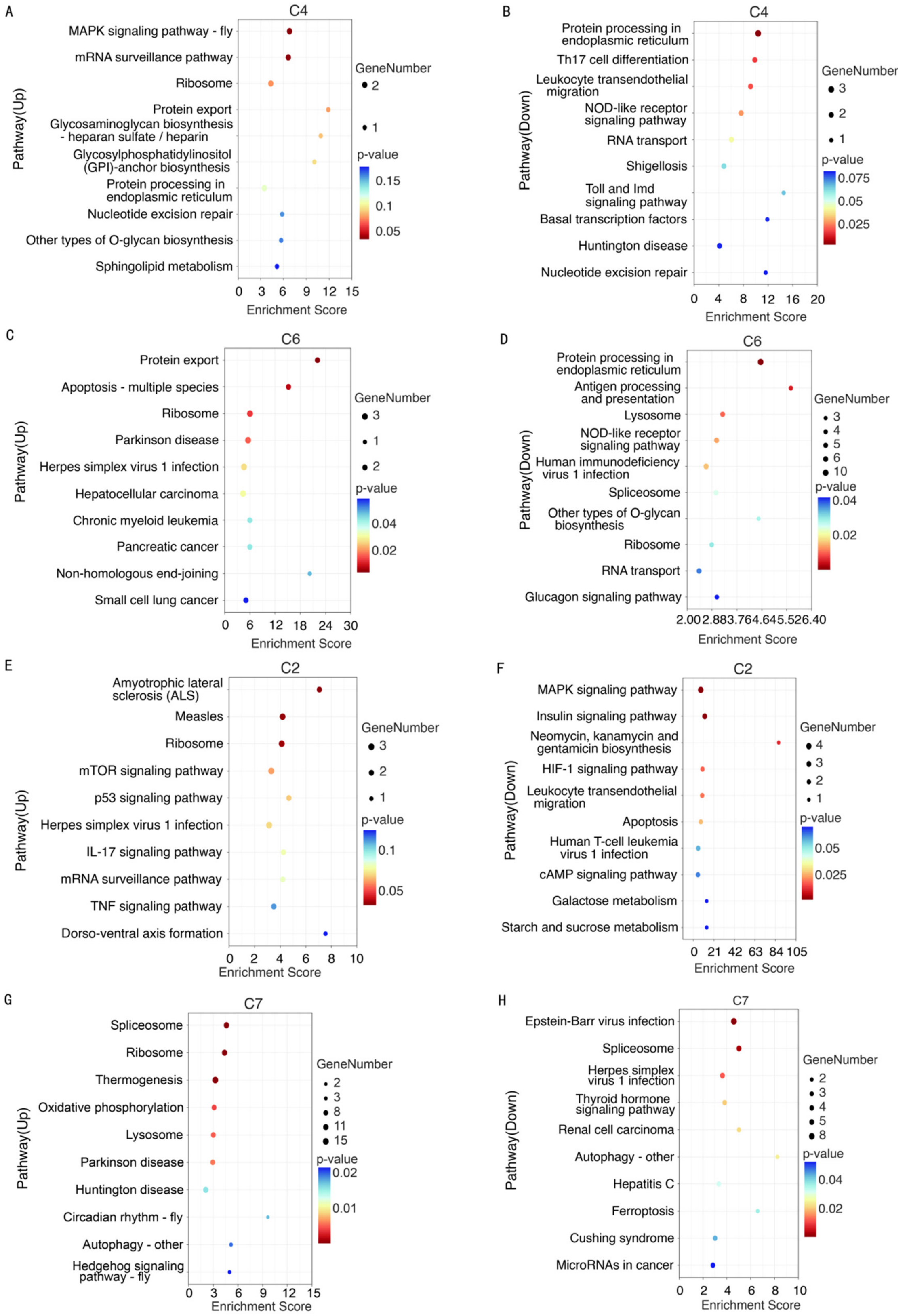

Figure S6. GO enrichment analysis（Up and Down）for chicken erythroid cell subpopulations of C4, C6, C2,C7.

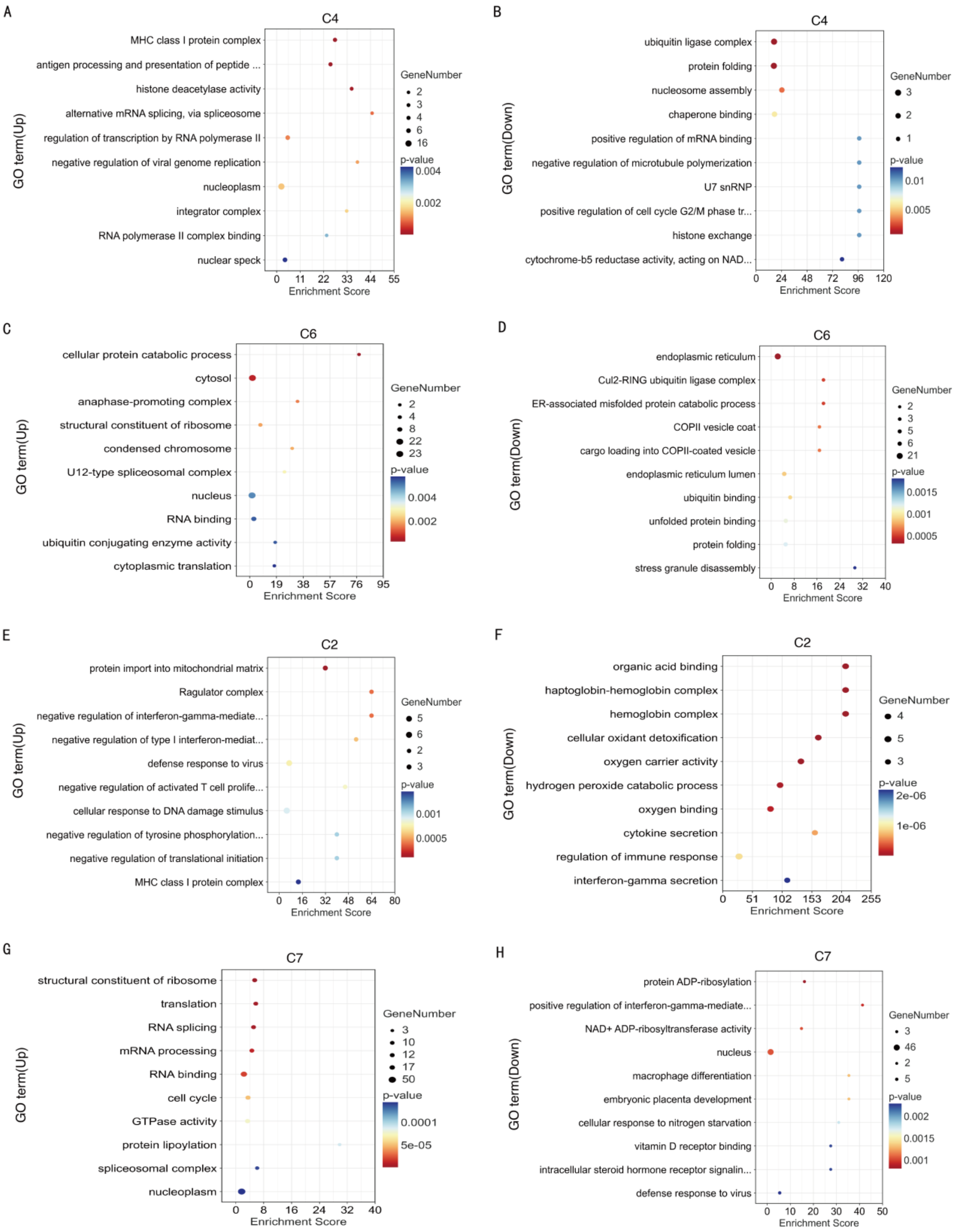

Figure S7. GO and KEGG enrichment analysis（Up and Down）for chicken erythroid cells bulk RNA-Seq DEGs.

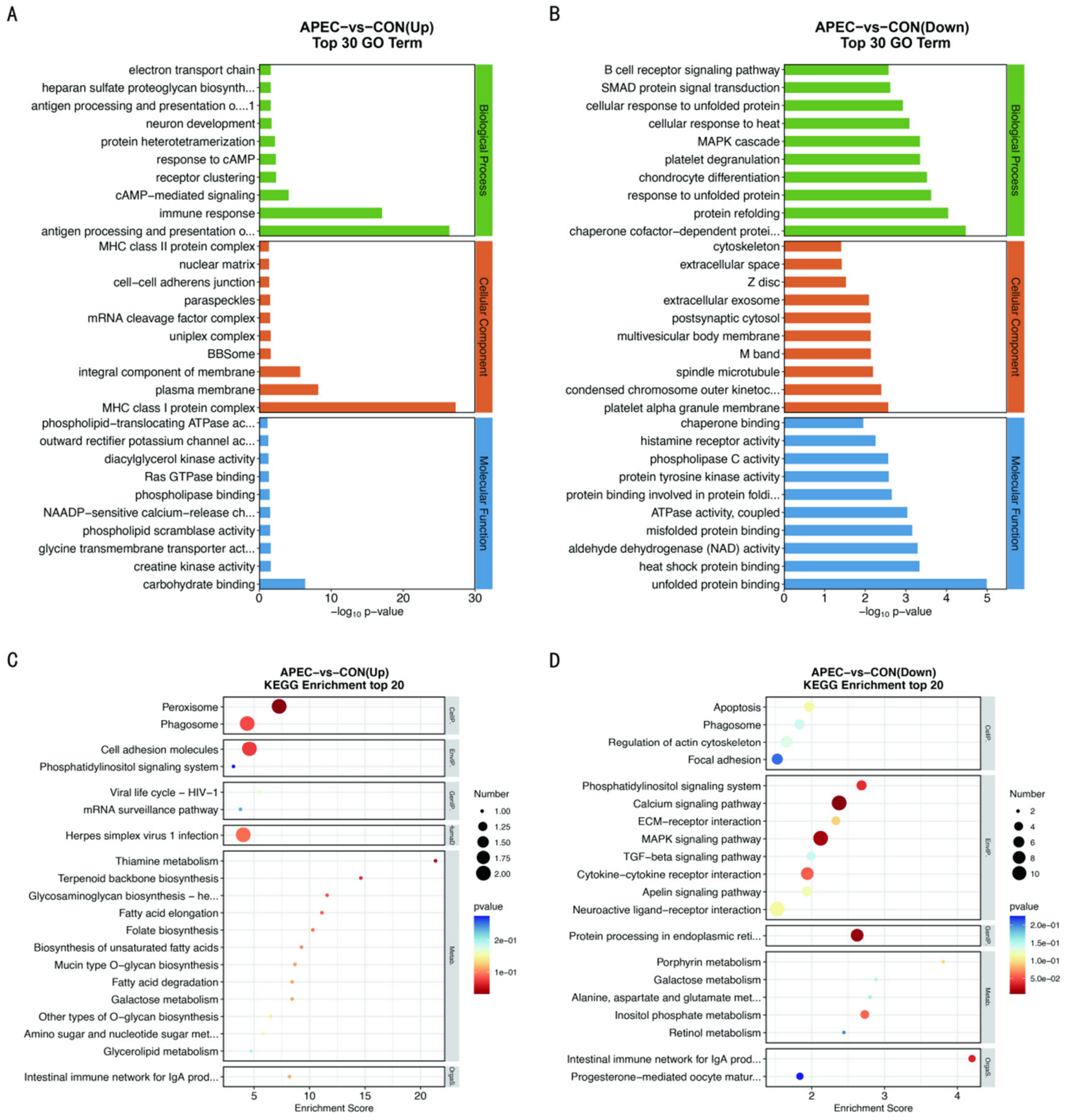

Figure S8. KEGG enrichment integrative analysis (up and down) for DEGs identified in chicken erythroid cells using Bulk-Seq and ScRNA-Seq methodologies (A-B). KEGG enrichment integrative analysis (up and down) for DEGs identified in chicken erythroid cells using subpopulations (C4, C6, C2, C7) and Bulk-Seq methodologies (C-D).

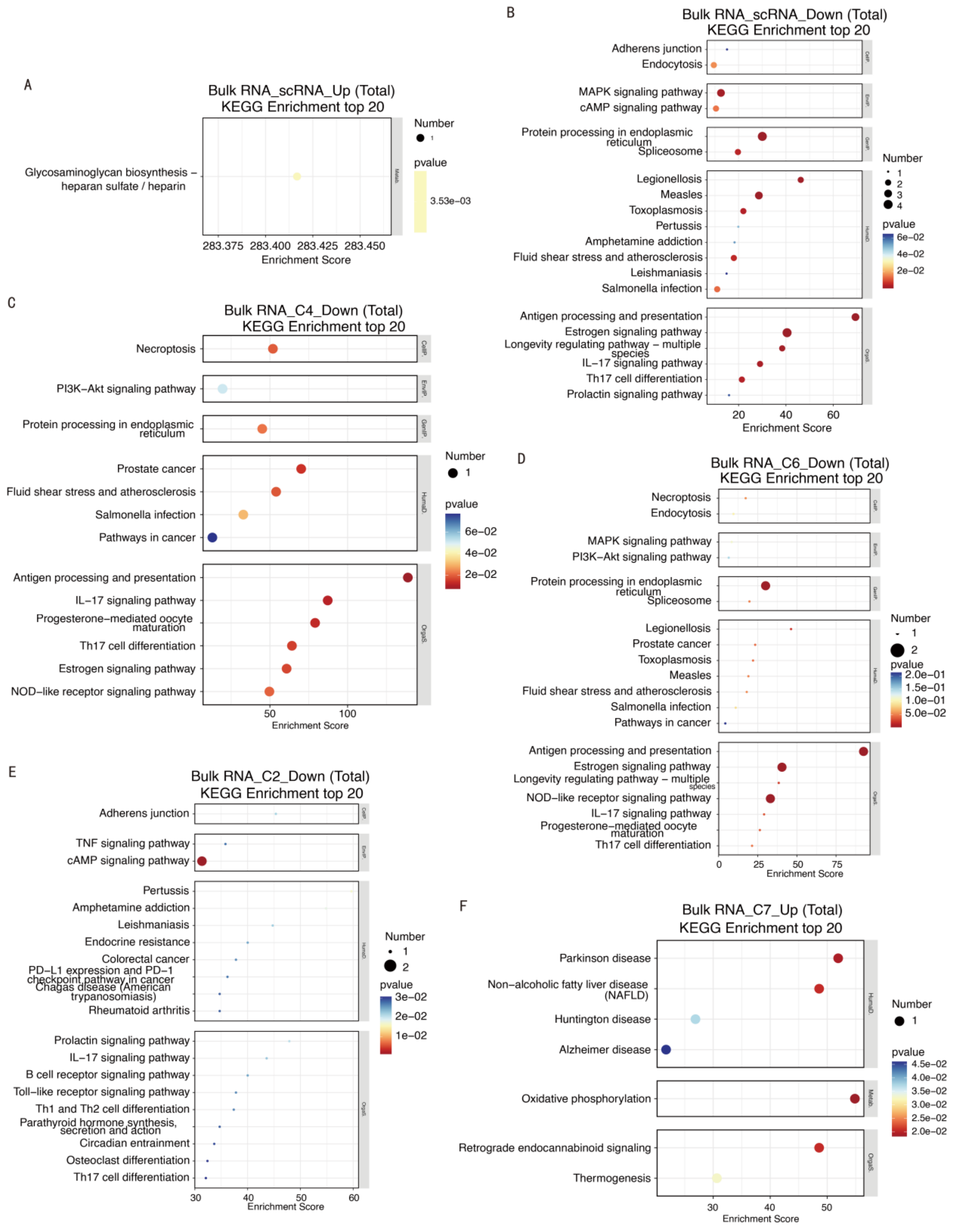

Supplement: Supplementary file 1 [file animals-16-00179-s001.zip › Content and figures-Supplementary materials.pdf]
